# Local genetic covariance analysis with lipid traits identifies novel loci for early-onset Alzheimer’s Disease

**DOI:** 10.1371/journal.pgen.1011631

**Published:** 2025-03-17

**Authors:** Nicholas R. Ray, Joseph Bradley, Elanur Yilmaz, Caghan Kizil, Jiji T. Kurup, Eden R. Martin, Hans-Ulrich Klein, Brian W. Kunkle, David A. Bennett, Philip L. De Jager, Alzheimer’s Disease Genetics Consortium, Gary W. Beecham, Carlos Cruchaga, Christiane Reitz

**Affiliations:** 1 Taub Institute for Research on Alzheimer’s Disease and the Aging Brain, Columbia University, New York, New York, United States of America; 2 Gertrude H. Sergievsky Center, Columbia University, New York, New York, United States of America; 3 NeuroGenomics and Informatics Center, Washington University School of Medicine in St. Louis, St. Louis, Missouri, United States of America; 4 The John P. Hussman Institute for Human Genomics, University of Miami, Miami, Florida, United States of America; 5 RUSH Alzheimer’s Disease Center, RUSH University, Chicago, Illinois, United States of America; University of North Carolina at Chapel Hill, UNITED STATES OF AMERICA

## Abstract

The genetic component of early-onset Alzheimer disease (EOAD), accounting for ~10% of all Alzheimer’s disease (AD) cases, is largely unexplained. Recent studies suggest that EOAD may be enriched for variants acting in the lipid pathway. The current study examines the shared genetic heritability between EOAD and the lipid pathway using genome-wide multi-trait genetic covariance analyses. Summary statistics were obtained from the GWAS meta-analyses of EOAD by the Alzheimer’s Disease Genetics Consortium (*n*=19,668) and five blood lipid traits by the Global Lipids Genetics Consortium (*n*=1,320,016). The significant results were compared between the EOAD and lipids GWAS and genetic covariance analyses were performed via SUPERGNOVA. Genes in linkage disequilibrium (LD) with top EOAD hits in identified regions of covariance with lipid traits were scored and ranked for causality by combining evidence from gene-based analysis, AD-risk scores incorporating transcriptomic and proteomic evidence, eQTL data, eQTL colocalization analyses, DNA methylation data, and single-cell RNA sequencing analyses. Direct comparison of GWAS results showed 5 loci overlapping between EOAD and at least one lipid trait harboring *APOE*, *TREM2*, *MS4A4E*, *LILRA5*, and *LRRC25*. Local genetic covariance analyses identified 3 regions of covariance between EOAD and at least one lipid trait. Gene prioritization nominated 3 likely causative genes at these loci: *ANKDD1B*, *CUZD1*, and *MS4A64*.The current study identified genetic covariance between EOAD and lipids, providing further evidence of shared genetic architecture and mechanistic pathways between the two traits.

## Author summary

Most individuals develop Alzheimer’s disease late in life, but approximately 10% of patients develop the disease before the age of 65 – these cases are known as early-onset Alzheimer’s disease (EOAD). Relatively little is known about the genes that contribute to non-autosomal dominant EOAD, but it is believed that some contributing genetic variants may be acting in the lipid pathway. We examined the genetic correlation between EOAD and five different lipid traits (total cholesterol, high-density lipoprotein cholesterol, low-density lipoprotein cholesterol, non-high-density lipoprotein cholesterol, and triglycerides) and identified three genomic regions that showed genetic overlap between EOAD and at least one of the five lipid traits. We used a novel gene prioritization approach that combined evidence from multiple sources to nominate three specific candidate genes in these regions: *ANKDD1B*, *CUZD1*, and *MS4A64*, all of which have been associated with lipid-related disorders and/or Alzheimer’s disease. Identification of mechanistic pathways shared between EOAD and lipid traits provides novel insights into the underlying etiology of these disorders and informs development of improved screening, prevention, and treatment strategies.

## Introduction

Alzheimer’s Disease (AD) is a highly prevalent progressive neurodegenerative disorder that places a substantial physical and emotional burden on patients and caregivers, and a significant financial toll on health care and social care systems [1]. While most AD patients are elderly individuals, 5-10% of cases occur before the age of 65 years and are classified as early-onset Alzheimer’s Disease (EOAD) [2]. EOAD has a substantial genetic basis with a heritability of 91% to 100% [3]. Studies of multiplex families with EOAD led to the identification of AD-causing mutations in the amyloid precursor protein (*APP*), presenilin 1 (*PSEN1*), and presenilin 2 (*PSEN2*) genes, playing a pivotal role in the implementation of the amyloid hypothesis in AD, which proposes an increase in β-amyloid production as a causative mechanism in AD etiology [4]. However, while the exact contribution of variation in *APP*, *PSEN1*, and *PSEN2* to EOAD prevalence is unknown, it is estimated to be less than 10% of all incident EOAD cases, leaving ~90% of EOAD cases unexplained [3–5]. A large proportion of EOAD heritability is expected to be explained by SNPs that do not pass the Bonferroni-corrected significance threshold [6].

**Fig 1:**
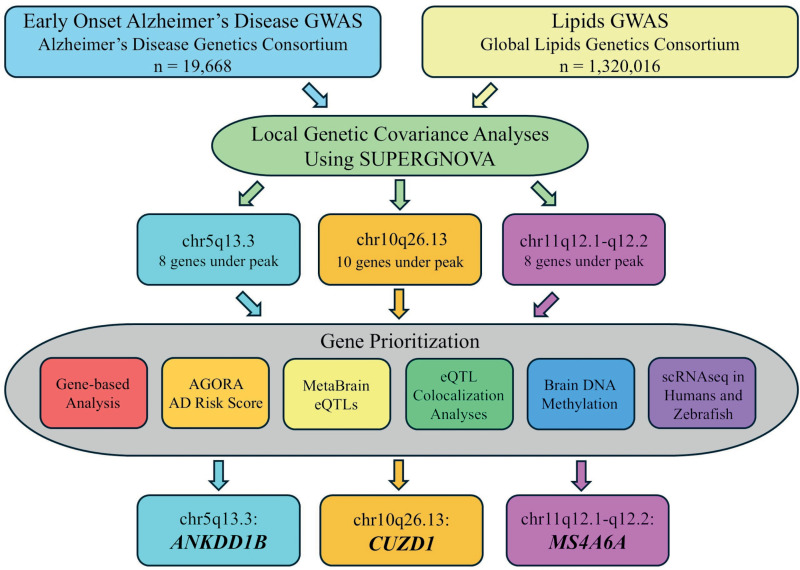
Flowchart showing input data (EOAD and Lipids GWAS) used in the primary analyses (local genetic covariance analysis using SUPERGNOVA). The primary analyses resulted in three regions of genetic covariance (chr5q13.3, chr10q26.13, and chr11q12.1-q12.2). The genes under the peak of the EOAD top hit in these regions were submitted to secondary analyses (gene-based analysis; look up of Agora AD risk scores; extraction of eQTL data and eQTL colocalization analyses; brain DNA methylation analysis; and single cell RNA sequencing analyses in humans and zebra fish). Composite scores were calculated for each gene based on the results of the secondary analyses and the top scoring gene for each region was *ANKDD1B* in chr5q13.3, *CUZD1* in chr10q26.13, and *MS4A6A* in chr11q12.1-q12.2.

Identification of the remaining genetic variation underlying EOAD and mapping of the mechanistic pathways involved is critical to disentangle the substantial clinical heterogeneity observed in this trait, develop prediction models, and develop more effective targets for screening, prevention, and treatment. A powerful approach to identify additional causative variants and biological pathways underlying complex traits are multi-trait analyses estimating local genetic covariance (i.e., genetic similarity in specific genomic regions) with other traits potentially sharing etiologic mechanisms. Acknowledging the importance of disentangling pleiotropy to pinpoint disease etiology and potentially reposition drugs for complex diseases, multi-trait modeling has recently undergone rapid developments and has resulted in significantly improved methods. To identify new genetic loci underlying EOAD, we examined genome-wide local genetic covariance with five lipid traits: total cholesterol (TC), high-density lipoprotein cholesterol (HDL-C), low-density lipoprotein cholesterol (LDL-C), non-high-density lipoprotein cholesterol (nonHDL-C), and triglycerides (TG). A large body of epidemiologic studies of AD by us [7,8] and others [9–15] has shown that cholesterol levels elevated in midlife increase the risk of AD and cognitive decline, and associations of higher LDL-C with increased cerebral β‐amyloid load have been observed in autopsy and in vivo imaging studies [16,17]. Similarly, AD risk is lower among statin users, and this association appears to be more pronounced with longer treatment exposure and the use of more potent drugs [18–23], although corresponding observational data on other lipid‐lowering drug classes are limited and ambiguous [24]. Studies specifically examining the association with EOAD are scarce, but a recent study of plasma samples from 2,125 EOAD cases and controls observed an association between EOAD and higher levels of LDL-C independent of the effects of *APOE*; as well as an enrichment of rare coding variants of *APOB*, a gene known to influence plasma cholesterol levels [25]. This supports the notion that EOAD may share common genetic loci with the lipid pathway. Following genetic covariance analyses, we validated putative loci observed to be shared between EOAD and any of the five lipid traits by conducting gene-based analysis; extracting publicly available AD risk scores calculated from genome-wide association studies (GWAS) and expression quantitative trait locus (eQTL), transcriptomic, and proteomic data; examining publicly available data from brain eQTL studies; performing colocalization analyses between EOAD summary statistics and eQTL data; inspecting brain DNA methylation data from the Religious Orders Study and Rush Memory and Aging Project (ROSMAP) cohorts; and analyzing single cell RNA sequencing data from both humans and zebrafish. See Fig 1 for an illustration of the methods and results.

## Results

All base-pair positions in this section refer to genome build GRCh37.

### Direct comparison of GWAS results

Direct comparison of GWAS results showed an overlap between EOAD and at least one of the lipid traits at five loci. One of these is the AD *APOE* risk haplotype on chr 19q13.32 (EOAD top SNP = rs59007384, *P* = 5.73×10^-311^) which also shows significant association with all four lipid traits (HDL top SNP = rs429358, *P* = 5.84×10^-256^; LDL top SNP = rs7412, *P* = 3.82×10^-305^; nonHDL top SNP = rs7412, *P* = 1.08×10^-305^; TC top SNP = rs7412, *P* = 1.98×10^-305^; TG top SNP = rs483082, *P* = 1.11×10^-305^) (see Fig A in S1 Text). In addition, the *TREM2* locus on chr6p21.1 (EOAD top SNP = rs75932628, *P* = 1.66×10^-15^) shows significant association with TG (rs742493, *P* = 2.68×10^-10^) (see Fig B in S1 Text). A locus at chr11q12.2 (EOAD top SNP = rs11824734; *P* = 2.88×10^-8^) is also significant for TC (rs950802; *P* = 4.64×10^-9^) (see Fig C in S1 Text). Based on gene prioritization analyses in the EOAD GWAS, the nearest gene to the variant is *MS4A6E*, while the prioritized gene at the locus is *MS4A4E*. Finally two independent additional loci on chr 19 show overlap: a locus on chr 19q13.42 harboring *LILRA5* (EOAD top SNP = rs2781753, *P* = 3.47×10^-10^) is also significantly associated with HDL (rs367070, *P* = 6.61×10^-140^), TC (rs1645788, *P* = 9.21×10^-21^), and TG (rs798889, *P* = 6.61×3.63×10^-10^) (see Fig D in S1 Text); and a locus on chr19p13.11 harboring *LRRC25* (EOAD top SNP = rs7258465, *P* = 8.91×10^-9^) is also associated with HDL (rs60570301, *P* = 1.73×10^-8^) (see Fig E in S1 Text).

### Analysis of genetic covariance between EOAD and lipid traits

Top results of the genetic covariance analyses with the five lipid traits (TC, HDL-C, LDL-C, nonHDL-C, and TG) are summarized in Table 1, with corresponding regional association plots displayed in Figs F-H in S1 Text. Genetic covariance analyses of EOAD with each of the five lipid traits identified 3 regions showing genetic correlation between EOAD and at least one of the five lipid traits at *P* < 2.12×10^-5^. The region showing strongest genetic correlation is located on chromosome 5q13.3 at 73,508,509-75,240,469 bp and showed genetic covariance between EOAD and TC (*P* = 5.5×10^-10^), LDL-C (*P* = 8.17×10^-9^), and nonHDL-C (*P* = 1.30×10^-10^) (Table 1 and Fig F in S1 Text). A second region on chromosome 10q26.13 at 123,855,124-124,894,743 bp showed genetic covariance between EOAD and TC (*P* = 3.78×10^-9^), LDL-C (*P* = 1.26×10^-8^), and nonHDL-C (*P* = 2.90×10^-9^) (Table 1 and Fig G in S1 Text). And lastly, a region on chromosome 11q12.1-q12.2 at 59,620,206-61,870,732 bp showed genetic covariance between EOAD and nonHDL-C (*P* = 1.60×10^-6^) (Table 1 and Fig H in S1 Text). This locus was also identified above in the direct comparison of GWAS results between EOAD and TC, and while the top SNP for nonHDL-C in the partition block assessed by SUPERGNOVA is located 1.5MB downstream of EOAD, there is also a suggestively significant set of SNPs in the same haplotype as the EOAD top hits (see Fig H-iii in S1 Text). Examination of LD patterns in these regions identified in total 26 genes under the peaks. A complete list of these genes in each region can be found in Table C in S1 Table.

**Table 1:** Results of genetic covariance analyses for regions with *P* < 2.12×10^-5^.

Chromosome	Start BP (GRCh37)	End BP (GRCh37)	Estimate of Covariance	Variance	*P*	# SNPs
**TC**						
5	73508509	75240469	0.002	9.46×10^-8^	5.50×10^-10^	3084
10	123855124	124894743	0.0007	1.39×10^-8^	3.78×10^-9^	2011
**LDL-C**						
5	73508509	75240469	0.002	1.07×10^-7^	8.17×10^-9^	3070
10	123855124	124894743	0.0007	1.60×10^-8^	1.26×10^-8^	2009
**nonHDL-C**						
5	73508509	75240469	0.002	7.32×10^-8^	1.30×10^-10^	3117
10	123855124	124894743	0.0006	1.18×10^-8^	2.90×10^-9^	2005
11	59620206	61870732	0.0006	1.47×10^-8^	1.60×10^-6^	3150

*abbreviations: TC = total Cholesterol; LDL-C = low-density lipoprotein cholesterol; nonHDL-C = non-high-density lipoprotein cholesterol; BP = base-pair position.

### Gene prioritization at identified loci showing genetic covariance

To prioritize genes in each of the 3 regions showing genetic covariance between EOAD and lipid traits, we calculated composite scores for each gene from gene-based analysis, Agora AD-risk scores, MetaBrain eQTL data, eQTL colocalization analyses, ROSMAP brain methylation data, and single cell RNA sequencing analyses in humans and zebrafish. Results from the gene-based analysis are displayed in Table D in S1 Table. Target Risk Scores extracted from Agora (https://agora.adknowledgeportal.org/) for each gene of interest are shown in Table E in S1 Table. Results from the MetaBrain cis-eQTL mapping for the 26 genes of interest with the minimum *P*-value for each gene are detailed in Tables F and G in S1 Table. Max H4 values from the coloc eQTL colocalization analyses were extracted for each gene of interest along with their associated eQTL datasets (Table H in S1 Table). The methylation site most significantly associated (minimum *P*-value) with both amyloid (Table I in S1 Table) and tau (Table J in S1 Table) pathology was extracted for each gene of interest. Results of the single-cell RNA sequencing analyses for humans and zebrafish, where a total score for each gene was derived by multiplying average proportion of expression by average level of expression by DEG index (see the Methods section for details), are detailed in Tables K and L in S1 Table.

**Fig 2:**
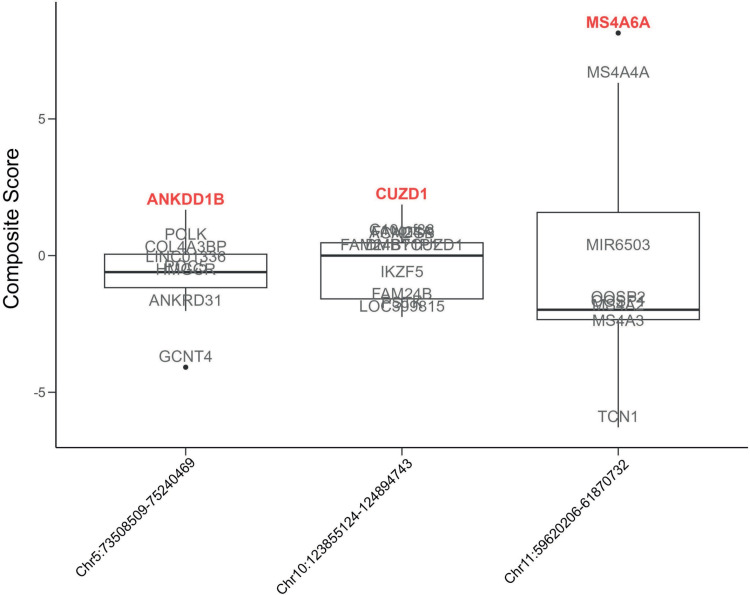
Boxplot of composite scores for all genes in each region resulting from the genetic covariance analyses between EOAD and lipid traits. The chromosome and base-pair start and end positions for each region are displayed on the x-axis using genome build GRCh37. The top scoring gene in each region is bolded and shown in red, while the rest of the genes are shown in grey.

Z-scores were calculated and summed for the variables mentioned above to create a composite score for each gene (Table M in S1 Table). Boxplots for these resulting scores for each significant region from the genetic covariance analyses are shown in Fig 2. Three genes were prioritized across the 3 regions: *ANKDD1B*, *CUZD1*, and *MS4A6A*.

*ANKDD1B* is the highest scoring gene in the region on chromosome 5q13.3 and is nominally associated with EOAD according to gene-based analysis (*P* = 2.9×10^-4^; Table D in S1 Table). Meta-analyses of *cis*-eQTLs for brain-related traits show at least one variant in the *ANKDD1B* gene to be highly significant in the cortex (*P* = 3.18×10^-110^; Table F in S1 Table) and nominally significant in the hippocampus (*P* =.003; Table G in S1 Table) [26]. *ANKDD1B* has an AD target risk score of 1.62 according to Agora (Table E in S1 Table), and colocalization analyses report a high probability that EOAD and eQTL data share a causal variant in the *ANKDD1B* gene (H4 =.82; Table H in S1 Table).

The highest scoring gene in the region on chromosome 10q26.13 is *CUZD1*. Gene-based analysis shows *CUZD1* to be nominally associated with EOAD (*P* =.01; Table D in S1 Table) and at least one variant in the *CUZD1* gene is a *cis*-eQTL in the cortex (*P* = 1.05×10^-91^;Table F in S1 Table) and the hippocampus (*P* = 1.94×10^-7^; Table G in S1 Table) [26]. Colocalization analyses report a high probability that EOAD and eQTL data share a causal variant in the *CUZD1* gene (H4 =.84; Table H in S1 Table) and Agora scores the AD risk of the gene at 1.48 (Table E in S1 Table).

Finally, the highest scoring gene in the region on chromosome 11q12.1-q12.2 is *MS4A6A*, which is in the same family as the *MS4A4E* gene reported in the direct comparison of GWAS results. *MS4A6A* is highly significant according to gene-based analysis (*P* = 4.07×10^-10^; Table D in S1 Table) and has a high AD risk score of 3.49 (Table E in S1 Table). At least one variant in *MS4A6A* is a significant eQTL in the cortex (*P* = 4.26×10^-6^; Table F in S1 Table) and the hippocampus (*P* = 6.15×10^-4^; Table G in S1 Table) [26], and EOAD is likely to share a causal variant with eQTLs in the gene according to colocalization analysis (H4 =.96; Table H in S1 Table). Analysis of brain methylation data report that at least one methylation site in *MS4A6A* is associated with both amyloid (*P* =.02; Table I in S1 Table) and tau (*P* =.005; Table J in S1 Table) pathology; and single cell RNA sequencing analysis shows that *MS4A6A* is differentially expressed between AD cases and controls in humans (*P* = 3.74×10^-20^; Table K in S1 Table).

## Discussion

To identify genetic regions and mechanistic pathways that are shared between early-onset EOAD and dyslipidemia, we examined shared significant loci between EOAD and five lipid trait GWAS and conducted hypothesis-free genetic covariance analyses between EOAD and the lipid traits. Five loci were reported in both the EOAD and lipid trait GWAS containing three well known AD risk genes – *APOE* [27], *TREM2* [28], *MS4A4E* [29] – as well as *LILRA5* and *LRRC25*. *LILRA5* belongs to a family of immunoglobulin-like receptors [30] and its expression is increased in the microglia of mice with amyloid plaques [31]. *LRRC25* encodes a leucine-rich repeat-containing protein and was identified as a potential AD risk gene in a recent study profiling chromatin accessibility in the microglia from 150 human brain samples [32].

The genetic covariance analyses identified 3 regions of genetic overlap on chromosomes 5q13.3, 10q26.13, and 11q12.1-q12.2. Construction of composite scores integrating gene-based analyses and a wealth of multi-omics data prioritized 3 genes in these regions most likely to be causal: *ANKDD1B*, *CUZD1*, and *MS4A6A*. All these identified genes act in mechanistic pathways related to AD and/or have been associated with AD related read-outs in animal, cell biological, or neuropathological studies.

*ANKDD1B*, the highest scoring gene in the region on chromosome 5q13.3, encodes the ankyrin repeat and death domain containing 1 B protein. *ANKDD1B* has been associated with dyslipidemia (specifically LDL-C) [33] and type 2 diabetes [34] in major GWAS studies, and is one of 28 genes included in a recent polygenic risk score for severe hypercholesterolemia (defined as LDL-C > 4.9 mmol/L) [35]. *ANKDD1B* was reported as one of two top genes connecting migraines and major depressive disorder in a genetic correlation analysis [36] and is hypermethylated in response to low-dose lead exposure in mice [37]. Diabetes, depression, and lead exposure have all been linked with cognitive decline and various neurological disorders including AD [38–43].

*CUZD1* is the highest scoring gene in the region on chromosome 10q26.13 and encodes a protein located in secretory granules in the pancreas that is thought to affect lipid-related metabolite levels [44]. *CUZD1* is a contributing gene to the zymogen activation pathway, which is enriched in the top 5% of genes associated with AD from a genome-wide meta-analysis [45]. Increased levels of the CUZD1 protein have also been correlated with genetic risk of migraine [46].

The highest scoring gene in the region on chromosome chr11q12.1-q12.2 is *MS4A6A*, a known AD risk gene that encodes a member of the membrane-spanning 4A gene family [47]. This locus is reported in the significant results from both the EOAD and lipids GWAS. Several meta-analyses have found that a variant (rs610932) in *MS4A6A* correlates with decreased risk for AD [47–49]. Colocalization analysis identified a shared causal variant in *MS4A6A* affecting a locus near *MS4A4A* in one of the most recent AD GWAS [50]. A transcriptome-wide association study found that increased expression of *MS4A6A* in monocytes associated with AD risk [50] and a DNA methylation study suggested that *MS4A6A* expression may mediate AD risk [51]. *MS4A6A* is also involved in the formation of atherosclerosis [52] and is differentially associated with various classes of lipids between AD cases and controls [53].

This study has several strengths. To our knowledge this is the first study assessing genetic covariance between the early-onset form of AD and the lipid pathway. AD cases and controls in the parent EOAD GWAS were derived from datasets with meticulous characterization for cognitive impairment, age at onset, and AD. In addition, to further validate shared identified loci, this study was able to capitalize on a variety of omics data from various independent sources, providing significant supportive evidence for the plausibility of candidate genes at identified loci. Several of the genes under consideration had data missing for at least one of the omics resources used in the prioritization score (see Tables D-M in S1 Table), so the results of this approach are limited by the availability of data on each gene. The evidence presented here is based on correlational analyses and functional follow-up for these genes is required to determine causality. Because we focus here on loci under the peak of the EOAD top hits in regions resulting from the genetic covariance analysis, we cannot exclude the possibility that we missed causal variants or cis-regulated genes operating outside of these loci. In addition, it is possible that regions of genetic covariance were missed in our study due to a lack of statistical power (particularly in the EOAD dataset) or lack of data from sex-stratiﬁed analyses.

In summary, this study suggests that EOAD shares genetic heritability with the lipids pathway, and that the common genetic loci include the *ANKDD1B, CUZD1,* and *MS4A6A* genes. These genes could lead to improved screening, prevention, and treatment for AD by targeting shared mechanistic pathways between EOAD and lipids. Future studies are needed that clarify the molecular mechanisms through which these genes modulate risk of EOAD, and that identify the specific causative variants at these and additional remaining loci that underlie the contribution of lipid metabolism to AD pathogenesis.

## Methods

### Ethics statement

This secondary data analysis of GWAS summary statistics was approved by the Columbia University Human Research Protection Office and Institutional Review Board (IRB #AAAQ9793).

### EOAD and lipid trait GWAS studies

Quality-controlled, ancestry-specific summary statistics were obtained from the GWAS meta-analyses of EOAD by the Alzheimer’s Disease Genetics Consortium (*n* = 19,668) and plasma lipid levels conducted by the Global Lipids Genetics Consortium (*n* = 1,320,016).

#### EOAD GWAS.

In brief, the EOAD GWAS included genetic data on 6,282 EOAD cases with AD diagnosis at or before age 70 and 13,386 cognitively normal controls with age at examination greater than 70 [54]. Participants were obtained from 40 independent datasets assembled through the Alzheimer’s Disease Genetics Consortium (ADGC), and European ancestry was determined by genetic principal component analysis. Demographic information for each cohort is described in Table A in S1 Table. Genetic data was genotyped on multiple genotyping arrays, QCed, imputed using the TOPMed imputation server, and aligned to the GRCh38 reference panel [54].

#### Lipids GWAS.

Data on the genetic architecture of lipid traits were derived from Graham et al. (2021) which meta-analyzed HDL-C, LDL-C, non-HDL-C, TC and TG summary statistics from 1,654,960 individuals across 201 individual studies [56]. Data from each cohort was QCed and imputed to the 1000 Genomes Phase 3 v5 (1KGP3) and the Haplotype Reference Consortium (HRC) panel [56,57]. The genetic covariance analyses presented here were conducted on ancestry-specific summary statistics obtained from 1,320,016 individuals of European ancestry.

### Direct comparison of GWAS results

We first conducted a direct comparison of association patterns in the EOAD and lipid trait GWAS data. For each genome-wide significant (*P* < 5×10^-8^) locus reported in the EOAD GWAS [54] a 1 MB region was created with 500kb to each side of the top SNP. Any genome-wide significant loci reported from the European-specific lipids GWAS [56] located within these regions were extracted and examined using locus zoom to confirm whether or not the haplotypes surrounding the top SNPs overlapped between EOAD and the respective lipid trait(s).

### Analysis of genetic covariance between EOAD and lipid traits

Estimation of genetic covariance of EOAD with individual lipid traits (TC, HDL-C, nonHDL-C, LDL-C, and TG) was performed via SUPERGNOVA [55]. SUPERGNOVA estimates local genetic correlation while taking into account linkage disequilibrium structure by decorrelating local z-scores with eigenvectors of the local LD matrix followed by estimation of local genetic covariance through a weighted least squares regression in each region. This technique has been demonstrated to be superior to other available methods such as LD score regression (LDSC) or GeNetic cOVariance Analyzer (GNOVA) [55]. The genome was partitioned into 2,353 approximately independent regions (~1.6 centimorgan on average) using LDetect [56], and LD was estimated using all European populations from the 1000 Genomes Phase3 reference panel [57]. Accordingly, the *P*-value threshold for significance of local genetic covariance between EOAD and each lipid trait was set based on a conservative Bonferroni threshold of *P* < 2.12×10^-5^ (0.05/2,353 bins). Regions resulting from the genetic covariance analyses were only reported if a strong genetic signal was observed in both traits, as defined by a *P*-value cutoff of 5×10^-5^. In addition, within-trait local genetic association and haplotype structure at these loci was assessed in the respective individual trait summary statistics (EOAD and the respective lipid trait showing genetic covariance), and visualized, annotated, and aligned across traits using LocusZoom [58].

### Gene prioritization

Genes included for prioritization were selected from the regions showing genetic correlation between EOAD and any of the five lipid traits. Locus zoom plots of these regions were inspected visually and any gene within the LD block of the EOAD top SNP in each region (any part of the gene can be within the LD block) was investigated further and given a composite score based on: 1) results from gene-based analysis; 2) AD risk scores from Agora (hosting high-dimensional human transcriptomic, proteomic, and metabolomic evidence for gene association with AD; 3) MetaBrain eQTL data; 4) eQTL colocalization analyses; 5) ROSMAP brain DNA methylation data (see below); and 6) single cell RNA sequencing data from both humans and zebrafish. The resulting composite scores were used to prioritize the top scoring gene in each region.

#### Gene-based analysis.

We performed gene-based analysis on our EOAD summary statistics using the MAGMA [59] software via the FUMA [60] web tool (https://fuma.ctglab.nl/). The 1000 Genomes Phase3 European reference panel [57] was employed along with the following parameters: FUMA v1.6.0; MAGMA v1.08; *P*-value of lead SNPs < 1×10^-5^; *P*-value of GWAS SNPs <.05; r^2^ threshold to define independent significant SNPs ≥ 0.6; second r^2^ threshold to define lead SNPs ≥ 0.1; minimum MAF = 0; maximum distance between LD blocks = 250kb. A window was set 35kb upstream and 10kb window downstream of the gene. 19,163 genes were tested by MAGMA, resulting in a Bonferroni-corrected *P*-value threshold of 2.61×10^-6^.

#### AD risk score data.

Genetic, multi-omic, and target AD risk scores were extracted for each gene of interest from the Agora tool (https://agora.adknowledgeportal.org/). The *Genetics Risk Score* ranges from 0-3 and is based on: 1) data from 24 GWAS or GWAS by proxy studies and 3 QTL studies; 2) phenotypic evidence supporting a gene from human and/or animal models; and 3) whether a gene has a model in development through the MODEL-AD consortium (https://www.model-ad.org/) [61]. The *Multi-omic Risk Score* ranges from 0-2 and is based on: 1) transcriptomic data from RNA-Seq profiling from 8 neocortical tissues and 2) proteomic data from label-free quantitation (LFQ) and Tandem Mass Tagging (TMT) shot-gun profiling methods generated from 8 neocortical tissues [61]. The *Target Risk Score* ranges from 0-5 and is a sum of the Genetic Risk Score and the Multi-omic Risk Score.

#### MetaBrain cis-eQTL data.

Results from *cis*-eQTL analyses from the cortex (n = 2,683) and hippocampus (n = 208) of individuals of European ancestry were obtained from MetaBrain (www.metabrain.nl). MetaBrain has collected 6,518 genotype samples and 8,613 bulk RNA-sequenced samples across 14 datasets and has performed ancestry and brain region specific *cis*- and *trans*-eQTL metanalyses [26], defining cis-eQTLs as common variants (MAF >1%) within 1 megabase (Mb) of the transcription start site of a protein-coding gene. For the present study, *cis*-eQTLs were extracted from this data for each gene under the peaks of the 3 regions resulting from the genetic covariance analyses, and the eQTL with the minimum *P*-value was selected to represent each gene.

#### eQTL colocalization analyses.

To calculate the probability that genes in identified regions of covariance between EOAD and at least one lipid trait share a single causal variant with eQTL loci (this probability is referred to as H4), we performed colocalization analyses between the regions resulting from our genetic covariance analyses and a set of 61 eQTL datasets (Table B in S1 Table) using the ‘coloc’ package in R [62–64].

#### Brain DNA methylation analyses in the ROS/MAP cohort.

DNA methylation data came from frozen dorsolateral prefrontal cortex from 761 participants in ROSMAP [65]. All ROSMAP participants enroll without known dementia, agree to annual clinical evaluation and agree to brain donation at the time of death. Both studies were approved by an Institutional Review Board of Rush University Medical Center. All participants signed informed and repository consents and an Anatomic Gift Act. Details of the data generation have been previously published, whereby methylation profiles were generated using the Illumina HumanMethylation450 beadset [66,67]. For the present analyses the β-values reported by the Illumina platform for each probe ranging from 0 (no methylation) to 1 (100% methylation) were utilized as the methylation level measurement for the targeted CG site in a given sample. To examine identified top loci for differentially methylated regions associated with AD pathology, we conducted a linear regression in R, adjusting for age at death, sex, experimental batch, and bisulfite conversion efficiency. β-amyloid load and PHF-tau tangle density were generated as previously described [68].

#### Single cell RNA sequencing.

To evaluate the RNA expressions of target genes at the single nucleus/cell level, we evaluated publicly available human single cell sequencing data (GSE157827) [69] and analyzed zebrafish data generated in-house. For human snRNA, we selected the single cell expression matrices of 4 AD and 4 control samples that were matched on sex and age. Matrices were generated with 10X function of the ‘Seurat’ (version 4.1.3) R package [70]. To create the Seurat object, we filtered out any cells with less than 200 expressed genes, and with genes expressed in less than 3 cells. Following the normalization of the dataset, the top 2,000 genes were used for further analyses. Anchors were identified with the *FindIntegrationAnchors* function and integration was performed using the *IntegrateData* function. We used ‘DoubletFinder’ [71] to remove doublets and performed the rest of the analyses on singlets only. The integrated Seurat object included 44,132 cells (27,198 for AD, and 16,934 for Control) with 29,772 features. The data were scaled using all genes, and 30 PCAs (RunPCA) were identified using the *RunPCA* function in the ‘Seurat’ package [70]. Thirty clusters were identified with resolution 1. The main cell types were defined using *AQP4* and *GFAP* for astroglia; *SLC17A7* and *NRGN* for excitatory neurons; *GAD1* and *GAD2* for inhibitory neurons; *PDGFRB*, *MCAM* and *GRM8* for pericytes; *C3* and *DOCK8* for microglia; *PLP1* and *MOBP* for oligodendrocytes; *PDGFRA* and *VCAN* for OPC; and *FLIT1* and *CLDN5* for endothelial cells.

We used the same methods and parameters as described above for creating a Seurat object with our zebrafish dataset. The main cell types were identified as described elsewhere [72,73]. Briefly, we used *s100b* and *gfap* for astroglia; *sv2a*, *nrgna*, *grin1a*, *grin1b* for neurons; *pdgfrb* and *kcne4* for pericytes; *cd74a* and *apoc1* for microglia; *mbpa* and *mpz* for oligodendrocytes; *aplnra* for OPC; *myh11a* and *tagln2* for vascular smooth muscle cells; *lyve1b* for lymph endothelial cells; and *kdrl* for vascular cells. To find the average expression of the genes, we used *AverageExpression* function. To define the percent expression of the given genes, *PrctCellExpringGene* was used.

Differential gene expression (DEG) in clusters was performed using the *FindMarkers* function by comparing AD cases against age-matched controls in humans and zebrafish injected with Aβ42 (AD model) versus those injected with phosphate buffered saline (control group). *P*-values and log2 fold change values of DEG results were transformed to generate the DEG index using the following equation: -log10(*P*) × (1 - |log2 fold change value|). If a gene is differentially expressed in multiple cell clusters, the one with lowest *P*-value was selected for the index. If no DEG was observed for a gene, a non-DEG penalty value of.25 was assigned for that gene.

#### Composite scores for each gene.

A single continuous value was either extracted or calculated for each of the types of evidence described above such that higher values generally indicate greater AD risk. *P*-values were extracted and transformed – using -log10(*P*) – from the results of the MetaBrain eQTL data, the gene-based analysis, and the ROSMAP methylation data for each gene. Where results existed for multiple variants or methylation probes within a gene, the variant with the minimum *P*-value was selected to represent the gene. The target risk score for each gene was extracted from Agora. After performing the colocalization analyses between the EOAD summary statistics and the multiple eQTL datasets, the maximum H4 value between EOAD and the various eQTL datasets was selected to represent each gene of interest. A score was calculated for each gene for both the human and zebrafish single-cell RNA sequencing analyses by multiplying the average proportion of each gene’s expression across different brain cell types by the average amount of each gene’s expression across the same cell types and by each gene’s DEG index. Z-scores were calculated for each of the variables described above, subsequently these z-scores were summed to form a total prioritization score for each gene. To standardize these variables, we calculated z-scores in R and then summed the z-scores to form a total prioritization score for each gene. The top scoring gene from each region resulting from the genetic covariance analyses was nominated as a priority gene for that locus.

## Supporting information

S1 Text Fig A. Locus zoom plots showing a 1MB region surrounding the EOAD top SNP chr19:45396665 and any overlapping genome-wide significant loci from the lipids GWAS. Fig B. Locus zoom plots showing a 1MB region surrounding the EOAD top SNP chr6:41129252 and any overlapping genome-wide significant loci from the lipids GWAS. Fig C. Locus zoom plots showing a 1MB region surrounding the EOAD top SNP chr11:60076693 and any overlapping genome-wide significant loci from the lipids GWAS. Fig D. Locus zoom plots showing a 1MB region surrounding the EOAD top SNP chr19:54814234 and any overlapping genome-wide significant loci from the lipids GWAS. Fig E. Locus zoom plots showing a 1MB region surrounding the EOAD top SNP chr19:18533642 and any overlapping genome-wide significant loci from the lipids GWAS. Fig F. Locus zoom plots for EOAD and each lipid trait that showed significant covariance with EOAD at chr5:73508509-75240469. Fig G. Locus zoom plots for EOAD and each lipid trait that showed significant covariance with EOAD at chr10:123855124-124894743. In Fig G-i the reference SNP was changed to second lowest P-value because the SNP with the lowest P-value did not appear in the 1000 Genomes reference panel and therefore did not show LD information. Fig H. Locus zoom plots for EOAD and each lipid trait that showed significant covariance with EOAD at chr11:59620206-61870732. Because of the extremely large effect shown to the right side of the nonHDL-C plot increasing the scale of the y-axis, we also include a plot for that trait zoomed into the region under the peak of the EOAD top hit.
(DOCX)

S1 Table Table A. Demographic information for each cohort included the EOAD GWAS on individuals of European ancestry. Table B. eQTL datasets used in colocalization analyses. Table C. Genes within the LD block of the EOAD top SNP for each region resulting from the genetic covariance analyses. Table D. Results of MAGMA gene-based analysis for genes in loci showing genetic correlation between EOAD and lipid traits. Table E. Agora AD risk scores for genes in loci showing genetic correlation between EOAD and lipid traits. Table F. Results from MetaBrain meta-analysis of cis-eQTLs in the cortex of Europeans for loci showing genetic correlation between EOAD and lipid traits. Table G. Results from MetaBrain meta-analysis of cis-eQTLs in the hippocampus of Europeans for loci showing genetic correlation between EOAD and lipid traits. Table H. Max H4 values from eQTL colocalization analyses for genes in loci showing genetic correlation between EOAD and lipid traits. Table I. Variants with most signficant (minimum P-value) amyloid methylation for each gene in loci showing genetic correlation between EOAD and lipid traits. Table J. Variants with most signficant (minimum P-value) tau methylation for each gene in loci showing genetic correlation between EOAD and lipid traits. Table K. Results of single cell RNA sequencing analyses in Humans for genes in loci showing genetic correlation between EOAD and lipid traits. Table L. Results of single cell RNA sequencing analyses in zebrafish for genes in loci showing genetic correlation between EOAD and lipid traits. Table M. Z-scores and composite scores for all variables used to calculate the composite scores for genes in loci showing genetic correlation between EOAD and lipid traits.
(XLSX)

## References

[1] Chemali Z, Withall A, Daffner KR. The plight of caring for young patients with frontotemporal dementia. Am J Alzheimers Dis Other Demen. 2010;25(2):109–15. doi: 10.1177/1533317509352335 20107238 PMC10845623

[2] Lambert MA, Bickel H, Prince M, Fratiglioni L, Von Strauss E, Frydecka D. Estimating the burden of early onset dementia; systematic review of disease prevalence. Eur J Neurol. 2014;21(4):563–9. 24418291 10.1111/ene.12325

[3] Wingo TS, Lah JJ, Levey AI, Cutler DJ. Autosomal recessive causes likely in early-onset Alzheimer disease. Arch Neurol. 2012;69(1):59–64. doi: 10.1001/archneurol.2011.221 21911656 PMC3332307

[4] Cacace R, Sleegers K, Van Broeckhoven C. Molecular genetics of early-onset Alzheimer’s disease revisited. Alzheimer’s & Dementia. 2016;12(6):733–48. 10.1016/j.jalz.2016.01.012 27016693

[5] Campion D, Dumanchin C, Hannequin D, Dubois B, Belliard S, Puel M, et al. Early-onset autosomal dominant Alzheimer disease: prevalence, genetic heterogeneity, and mutation spectrum. Am J Hum Genet. 1999;65(3):664–70. doi: 10.1086/302553 10441572 PMC1377972

[6] Yang J, Benyamin B, McEvoy BP, Gordon S, Henders AK, Nyholt DR, et al. Common SNPs explain a large proportion of the heritability for human height. Nat Genet. 2010;42(7):565–9. doi: 10.1038/ng.608 20562875 PMC3232052

[7] Reitz C, Tang M-X, Schupf N, Manly JJ, Mayeux R, Luchsinger JA. Association of higher levels of high-density lipoprotein cholesterol in elderly individuals and lower risk of late-onset Alzheimer disease. Arch Neurol. 2010;67(12):1491–7. doi: 10.1001/archneurol.2010.297 21149810 PMC3065942

[8] Reitz C, Tang M-X, Luchsinger J, Mayeux R. Relation of plasma lipids to Alzheimer disease and vascular dementia. Arch Neurol. 2004;61(5):705–14. doi: 10.1001/archneur.61.5.705 15148148 PMC2696387

[9] Kivipelto M, Solomon A. Cholesterol as a risk factor for Alzheimer’s disease - epidemiological evidence. Acta Neurol Scand Suppl. 2006;185:50–7. doi: 10.1111/j.1600-0404.2006.00685.x 16866911

[10] Shepardson NE, Shankar GM, Selkoe DJ. Cholesterol level and statin use in Alzheimer disease: I. Review of epidemiological and preclinical studies. Arch Neurol. 2011;68(10):1239–44. doi: 10.1001/archneurol.2011.203 21987540 PMC3211071

[11] Kivipelto M, Helkala E-L, Laakso MP, Hänninen T, Hallikainen M, Alhainen K, et al. Apolipoprotein E epsilon4 allele, elevated midlife total cholesterol level, and high midlife systolic blood pressure are independent risk factors for late-life Alzheimer disease. Ann Intern Med. 2002;137(3):149–55. doi: 10.7326/0003-4819-137-3-200208060-00006 12160362

[12] Notkola IL, Sulkava R, Pekkanen J, Erkinjuntti T, Ehnholm C, Kivinen P, et al. Serum total cholesterol, apolipoprotein E epsilon 4 allele, and Alzheimer’s disease. Neuroepidemiology. 1998;17(1):14–20. doi: 10.1159/000026149 9549720

[13] Kalmijn S, Foley D, White L, Burchfiel CM, Curb JD, Petrovitch H, et al. Metabolic cardiovascular syndrome and risk of dementia in Japanese-American elderly men. The Honolulu-Asia aging study. Arterioscler Thromb Vasc Biol. 2000;20(10):2255–60. doi: 10.1161/01.atv.20.10.2255 11031212

[14] Whitmer RA, Sidney S, Selby J, Johnston SC, Yaffe K. Midlife cardiovascular risk factors and risk of dementia in late life. Neurology. 2005;64(2):277–81. doi: 10.1212/01.WNL.0000149519.47454.F2 15668425

[15] Jarvik GP, Wijsman EM, Kukull WA, Schellenberg GD, Yu C, Larson EB. Interactions of apolipoprotein E genotype, total cholesterol level, age, and sex in prediction of Alzheimer’s disease: a case-control study. Neurology. 1995;45(6):1092–6. doi: 10.1212/wnl.45.6.1092 7783869

[16] Matsuzaki T, Sasaki K, Hata J, Hirakawa Y, Fujimi K, Ninomiya T, et al. Association of Alzheimer disease pathology with abnormal lipid metabolism: the Hisayama Study. Neurology. 2011;77(11):1068–75. doi: 10.1212/WNL.0b013e31822e145d 21911734

[17] Reed B, Villeneuve S, Mack W, DeCarli C, Chui HC, Jagust W. Associations between serum cholesterol levels and cerebral amyloidosis. JAMA Neurol. 2014;71(2):195–200. doi: 10.1001/jamaneurol.2013.5390 24378418 PMC4083819

[18] Wolozin B, Kellman W, Ruosseau P, Celesia GG, Siegel G. Decreased prevalence of Alzheimer disease associated with 3-hydroxy-3-methyglutaryl coenzyme A reductase inhibitors. Arch Neurol. 2000;57(10):1439–43. doi: 10.1001/archneur.57.10.1439 11030795

[19] Li G, Larson EB, Sonnen JA, Shofer JB, Petrie EC, Schantz A, et al. Statin therapy is associated with reduced neuropathologic changes of Alzheimer disease. Neurology. 2007;69(9):878–85. doi: 10.1212/01.wnl.0000277657.95487.1c 17724290

[20] Jick H, Zornberg GL, Jick SS, Seshadri S, Drachman DA. Statins and the risk of dementia. Lancet. 2000;356(9242):1627–31. doi: 10.1016/s0140-6736(00)03155-x 11089820

[21] Sparks DL, Kryscio RJ, Sabbagh MN, Connor DJ, Sparks LM, Liebsack C. Reduced risk of incident AD with elective statin use in a clinical trial cohort. Curr Alzheimer Res. 2008;5(4):416–21. doi: 10.2174/156720508785132316 18690839

[22] Cramer C, Haan MN, Galea S, Langa KM, Kalbfleisch JD. Use of statins and incidence of dementia and cognitive impairment without dementia in a cohort study. Neurology. 2008;71(5):344–50. doi: 10.1212/01.wnl.0000319647.15752.7b 18663180 PMC2676946

[23] Haag MDM, Hofman A, Koudstaal PJ, Stricker BHC, Breteler MMB. Statins are associated with a reduced risk of Alzheimer disease regardless of lipophilicity. The Rotterdam Study. J Neurol Neurosurg Psychiatry. 2009;80(1):13–7. doi: 10.1136/jnnp.2008.150433 18931004

[24] Larsson SC, Markus HS. Does Treating Vascular Risk Factors Prevent Dementia and Alzheimer’s Disease? A Systematic Review and Meta-Analysis. J Alzheimers Dis. 2018;64(2):657–68. 29914039 10.3233/JAD-180288

[25] Wingo TS, Cutler DJ, Wingo AP, Le N-A, Rabinovici GD, Miller BL, et al. Association of Early-Onset Alzheimer Disease With Elevated Low-Density Lipoprotein Cholesterol Levels and Rare Genetic Coding Variants of APOB. JAMA Neurol. 2019;76(7):809–17. doi: 10.1001/jamaneurol.2019.0648 31135820 PMC6547122

[26] de Klein N, Tsai EA, Vochteloo M, Baird D, Huang Y, Chen C-Y, et al. Brain expression quantitative trait locus and network analyses reveal downstream effects and putative drivers for brain-related diseases. Nat Genet. 2023;55(3):377–88. doi: 10.1038/s41588-023-01300-6 36823318 PMC10011140

[27] Mayeux R, Stern Y, Ottman R, Tatemichi TK, Tang MX, Maestre G, et al. The apolipoprotein epsilon 4 allele in patients with Alzheimer’s disease. Ann Neurol. 1993;34(5):752–4. doi: 10.1002/ana.410340527 8239575

[28] Guerreiro R, Wojtas A, Bras J, Carrasquillo M, Rogaeva E, Majounie E, et al. TREM2 variants in Alzheimer’s disease. N Engl J Med. 2013;368(2):117–27. doi: 10.1056/NEJMoa1211851 23150934 PMC3631573

[29] Hollingworth P, Harold D, Sims R, Gerrish A, Lambert J-C, Carrasquillo MM, et al. Common variants at ABCA7, MS4A6A/MS4A4E, EPHA1, CD33 and CD2AP are associated with Alzheimer’s disease. Nat Genet. 2011;43(5):429–35. doi: 10.1038/ng.803 21460840 PMC3084173

[30] Borges L, Kubin M, Kuhlman T. LIR9, an immunoglobulin-superfamily-activating receptor, is expressed as a transmembrane and as a secreted molecule. Blood. 2003;101(4):1484–6. doi: 10.1182/blood-2002-05-1432 12393390

[31] Salih DA, Bayram S, Guelfi S, Reynolds RH, Shoai M, Ryten M, et al. Genetic variability in response to amyloid beta deposition influences Alzheimer’s disease risk. Brain Commun. 2019;1(1):fcz022. doi: 10.1093/braincomms/fcz022 32274467 PMC7145452

[32] Kosoy R, Fullard JF, Zeng B, Bendl J, Dong P, Rahman S, et al. Genetics of the human microglia regulome refines Alzheimer’s disease risk loci. Nat Genet. 2022;54(8):1145–54. doi: 10.1038/s41588-022-01149-1 35931864 PMC9388367

[33] Kang M, Sung J. A genome-wide search for gene-by-obesity interaction loci of dyslipidemia in Koreans shows diverse genetic risk alleles. J Lipid Res. 2019;60(12):2090–101. doi: 10.1194/jlr.P119000226 31662442 PMC6889708

[34] Vujkovic M, Keaton J, Lynch J, Miller D, Zhou J, Tcheandjieu C. Discovery of 318 new risk loci for type 2 diabetes and related vascular outcomes among 1.4 million participants in a multi-ancestry meta-analysis. Nature Genetics. 2020;52(7):680–91. doi: 10.1038/s41588-020-00776-6 32541925 PMC7343592

[35] Leal LG, Hoggart C, Jarvelin M-R, Herzig K-H, Sternberg MJE, David A. A polygenic biomarker to identify patients with severe hypercholesterolemia of polygenic origin. Mol Genet Genomic Med. 2020;8(6):e1248. doi: 10.1002/mgg3.1248 32307928 PMC7284038

[36] Yang Y, Zhao H, Boomsma DI, Ligthart L, Belin AC, Smith GD, et al. Molecular genetic overlap between migraine and major depressive disorder. Eur J Hum Genet. 2018;26(8):1202–16. doi: 10.1038/s41431-018-0150-2 29995844 PMC6057914

[37] Dou JF, Farooqui Z, Faulk CD, Barks AK, Jones T, Dolinoy DC, et al. Perinatal Lead (Pb) Exposure and Cortical Neuron-Specific DNA Methylation in Male Mice. Genes (Basel). 2019;10(4). 10.3390/genes10040274 PMC6523909 30987383

[38] Green RC, Cupples LA, Kurz A, Auerbach S, Go R, Sadovnick D, et al. Depression as a risk factor for Alzheimer disease: the MIRAGE Study. Arch Neurol. 2003;60(5):753–9. doi: 10.1001/archneur.60.5.753 12756140

[39] Herbert J, Lucassen PJ. Depression as a risk factor for Alzheimer’s disease: Genes, steroids, cytokines and neurogenesis - What do we need to know?. Front Neuroendocrinol. 2016;41:153–71. doi: 10.1016/j.yfrne.2015.12.001 26746105

[40] Kopf D, Frölich L. Risk of incident Alzheimer’s disease in diabetic patients: a systematic review of prospective trials. Journal of Alzheimer’s Disease. 2009;16(4):677–85. 10.3233/JAD-2009-1011 19387104

[41] Mittal K, Katare DP. Shared links between type 2 diabetes mellitus and Alzheimer’s disease: A review. Diabetes Metab Syndr. 2016;10(2 Suppl 1):S144–9. doi: 10.1016/j.dsx.2016.01.021 26907971

[42] Tasin FR, Ahmed A, Halder D, Mandal C. On-going consequences of in utero exposure of Pb: An epigenetic perspective. J Appl Toxicol. 2022;42(10):1553–69. doi: 10.1002/jat.4287 35023172

[43] Xia M, Yang L, Sun G, Qi S, Li B. Mechanism of depression as a risk factor in the development of Alzheimer’s disease: the function of AQP4 and the glymphatic system. Psychopharmacology (Berl). 2017;234(3):365–79. doi: 10.1007/s00213-016-4473-9 27837334

[44] Feofanova EV, Brown MR, Alkis T, Manuel AM, Li X, Tahir UA. Whole-genome sequencing analysis of human metabolome in multi-ethnic populations. Nature Communications. 2023;14(1):3111. 10.1038/s41467-023-38800-2 PMC10229598 37253714

[45] Hong M-G, Reynolds CA, Feldman AL, Kallin M, Lambert J-C, Amouyel P, et al. Genome-wide and gene-based association implicates FRMD6 in Alzheimer disease. Hum Mutat. 2012;33(3):521–9. doi: 10.1002/humu.22009 22190428 PMC3326347

[46] Tanha HM, International Headache Genetics Consortium, Nyholt DR. Genetic analyses identify pleiotropy and causality for blood proteins and highlight Wnt/β-catenin signalling in migraine. Nat Commun. 2022;13(1):2593. doi: 10.1038/s41467-022-30184-z 35546551 PMC9095680

[47] Almeida JFF, Dos Santos LR, Trancozo M, de Paula F. Updated Meta-Analysis of BIN1, CR1, MS4A6A, CLU, and ABCA7 Variants in Alzheimer’s Disease. J Mol Neurosci. 2018;64(3):471–7. doi: 10.1007/s12031-018-1045-y 29504051

[48] Ji W, Xu L, Zhou H, Wang S, Fang Y. Meta-analysis of association between the genetic polymorphisms on chromosome 11q and Alzheimer’s disease susceptibility. Int J Clin Exp Med. 2015;8(10):18235–44. 26770425 PMC4694325

[49] Mao Y-F, Guo Z-Y, Pu J-L, Chen Y-X, Zhang B-R. Association of CD33 and MS4A cluster variants with Alzheimer’s disease in East Asian populations. Neurosci Lett. 2015;609:235–9. doi: 10.1016/j.neulet.2015.10.007 26455864

[50] Harwood JC, Leonenko G, Sims R, Escott-Price V, Williams J, Holmans P. Defining functional variants associated with Alzheimer’s disease in the induced immune response. Brain Commun. 2021;3(2):fcab083. doi: 10.1093/braincomms/fcab083 33959712 PMC8087896

[51] Sun Y, Zhu J, Yang Y, Zhang Z, Zhong H, Zeng G, et al. Identification of candidate DNA methylation biomarkers related to Alzheimer’s disease risk by integrating genome and blood methylome data. Transl Psychiatry. 2023;13(1):387. doi: 10.1038/s41398-023-02695-w 38092781 PMC10719322

[52] Chen Y, You N, Yang C, Zhang J. Helicobacter pylori infection increases the risk of carotid plaque formation: Clinical samples combined with bioinformatics analysis. Heliyon. 2023;9(9):e20037. doi: 10.1016/j.heliyon.2023.e20037 37809782 PMC10559771

[53] Liu Y, Thalamuthu A, Mather KA, Crawford J, Ulanova M, Wong MWK, et al. Plasma lipidome is dysregulated in Alzheimer’s disease and is associated with disease risk genes. Transl Psychiatry. 2021;11(1):344. doi: 10.1038/s41398-021-01362-2 34092785 PMC8180517

[54] Cruchaga C, Bradley J, Western D, Wang C, Lucio Da Fonseca E, Neupane A, et al. Novel early-onset Alzheimer-associated genes influence risk through dysregulation of glutamate, immune activation, and intracell signaling pathways. Res Sq. 2024. 10.1002/alz.70377 PMC12187977 40556318

[55] Zhang Y, Lu Q, Ye Y, Huang K, Liu W, Wu Y, et al. SUPERGNOVA: local genetic correlation analysis reveals heterogeneous etiologic sharing of complex traits. Genome Biol. 2021;22(1):262. doi: 10.1186/s13059-021-02478-w 34493297 PMC8422619

[56] Berisa T, Pickrell JK. Approximately independent linkage disequilibrium blocks in human populations. Bioinformatics. 2016;32(2):283–5. doi: 10.1093/bioinformatics/btv546 26395773 PMC4731402

[57] 1000 Genomes Project Consortium, Abecasis GR, Auton A, Brooks LD, DePristo MA, Durbin RM, et al. An integrated map of genetic variation from 1,092 human genomes. Nature. 2012;491(7422):56–65. doi: 10.1038/nature11632 23128226 PMC3498066

[58] Pruim RJ, Welch RP, Sanna S, Teslovich TM, Chines PS, Gliedt TP, et al. LocusZoom: regional visualization of genome-wide association scan results. Bioinformatics. 2010;26(18):2336–7. doi: 10.1093/bioinformatics/btq419 20634204 PMC2935401

[59] de Leeuw CA, Mooij JM, Heskes T, Posthuma D. MAGMA: generalized gene-set analysis of GWAS data. PLoS Comput Biol. 2015;11(4):e1004219. doi: 10.1371/journal.pcbi.1004219 25885710 PMC4401657

[60] Watanabe K, Taskesen E, van Bochoven A, Posthuma D. Functional mapping and annotation of genetic associations with FUMA. Nat Commun. 2017;8(1):1826. doi: 10.1038/s41467-017-01261-5 29184056 PMC5705698

[61] Cary G, Wiley J, Gockley J, Keegan S, Heath L, Butler III R. Genetic and multi-omic risk assessment of Alzheimer’s disease implicates core associated biological domains. medRxiv. 2022. doi: 10.1101/2022.12.15.22283478 PMC11033838 38650747

[62] Giambartolomei C, Vukcevic D, Schadt EE, Franke L, Hingorani AD, Wallace C, et al. Bayesian test for colocalisation between pairs of genetic association studies using summary statistics. PLoS Genet. 2014;10(5):e1004383. doi: 10.1371/journal.pgen.1004383 24830394 PMC4022491

[63] R Core Team R. R: A language and environment for statistical computing. 2013.

[64] Wallace C. Eliciting priors and relaxing the single causal variant assumption in colocalisation analyses. PLoS Genet. 2020;16(4):e1008720. doi: 10.1371/journal.pgen.1008720 32310995 PMC7192519

[65] Bennett DA, Buchman AS, Boyle PA, Barnes LL, Wilson RS, Schneider JA. Religious Orders Study and Rush Memory and Aging Project. J Alzheimers Dis. 2018;64(s1):S161–89. doi: 10.3233/JAD-179939 29865057 PMC6380522

[66] Bennett D, Yu L, De Jager P. Building a pipeline to discover and validate novel therapeutic targets and lead compounds for Alzheimer’s disease. Biochem Pharmacol. 2014;88(4):617–30. 24508835 10.1016/j.bcp.2014.01.037 PMC4054869

[67] De Jager PL, Ma Y, McCabe C, Xu J, Vardarajan BN, Felsky D, et al. A multi-omic atlas of the human frontal cortex for aging and Alzheimer’s disease research. Sci Data. 2018;5:180142. doi: 10.1038/sdata.2018.142 30084846 PMC6080491

[68] Wilson RS, Arnold SE, Schneider JA, Tang Y, Bennett DA. The relationship between cerebral Alzheimer’s disease pathology and odour identification in old age. J Neurol Neurosurg Psychiatry. 2007;78(1):30–5. doi: 10.1136/jnnp.2006.099721 17012338 PMC2117790

[69] Lau S-F, Cao H, Fu AKY, Ip NY. Single-nucleus transcriptome analysis reveals dysregulation of angiogenic endothelial cells and neuroprotective glia in Alzheimer’s disease. Proc Natl Acad Sci U S A. 2020;117(41):25800–9. doi: 10.1073/pnas.2008762117 32989152 PMC7568283

[70] Stuart T, Butler A, Hoffman P, Hafemeister C, Papalexi E, Mauck WM 3rd, et al. Comprehensive Integration of Single-Cell Data. Cell. 2019;177(7):1888–1902.e21. doi: 10.1016/j.cell.2019.05.031 31178118 PMC6687398

[71] McGinnis CS, Murrow LM, Gartner ZJ. DoubletFinder: Doublet Detection in Single-Cell RNA Sequencing Data Using Artificial Nearest Neighbors. Cell Syst. 2019;8(4):329–337.e4. doi: 10.1016/j.cels.2019.03.003 30954475 PMC6853612

[72] Cosacak MI, Bhattarai P, De Jager PL, Menon V, Tosto G, Kizil C. Single Cell/Nucleus Transcriptomics Comparison in Zebrafish and Humans Reveals Common and Distinct Molecular Responses to Alzheimer’s Disease. Cells. 2022;11(11):1807. doi: 10.3390/cells11111807 35681503 PMC9180693

[73] Cosacak MI, Bhattarai P, Reinhardt S, Petzold A, Dahl A, Zhang Y, et al. Single-Cell Transcriptomics Analyses of Neural Stem Cell Heterogeneity and Contextual Plasticity in a Zebrafish Brain Model of Amyloid Toxicity. Cell Rep. 2019;27(4):1307-1318.e3. doi: 10.1016/j.celrep.2019.03.090 31018142

[74] Graham SE, Clarke SL, Wu K-HH, Kanoni S, Zajac GJM, Ramdas S, et al. The power of genetic diversity in genome-wide association studies of lipids. Nature. 2021;600(7890):675–9. doi: 10.1038/s41586-021-04064-3 34887591 PMC8730582

