# Supplementary material for: Local genetic covariance analysis with lipid traits identifies novel loci for early-onset Alzheimer’s Disease
Source: PLoS Genet. 2025 Mar 17;21(3):e1011631. doi: 10.1371/journal.pgen.1011631 (PMC11984970; doi:10.1371/journal.pgen.1011631)

**S1 Text**

Contents

[Fig A. Locus zoom plots showing a 1MB region surrounding the EOAD top SNP chr19:45396665 and any overlapping genome-wide significant loci from the lipids GWAS. 2](#_Toc185500149)

[Fig B. Locus zoom plots showing a 1MB region surrounding the EOAD top SNP chr6:41129252 and any overlapping genome-wide significant loci from the lipids GWAS. 8](#_Toc185500150)

[Fig C. Locus zoom plots showing a 1MB region surrounding the EOAD top SNP chr11:60076693 and any overlapping genome-wide significant loci from the lipids GWAS. 10](#_Toc185500151)

[Fig D. Locus zoom plots showing a 1MB region surrounding the EOAD top SNP chr19:54814234 and any overlapping genome-wide significant loci from the lipids GWAS. 12](#_Toc185500152)

[Fig E. Locus zoom plots showing a 1MB region surrounding the EOAD top SNP chr19:18533642 and any overlapping genome-wide significant loci from the lipids GWAS. 16](#_Toc185500153)

[Fig F. Locus zoom plots for EOAD and each lipid trait that showed significant covariance with EOAD at chr5:73508509-75240469. 18](#_Toc185500154)

[Fig G. Locus zoom plots for EOAD and each lipid trait that showed significant covariance with EOAD at chr10:123855124-124894743. 22](#_Toc185500155)

[Fig H. Locus zoom plots for EOAD and each lipid trait that showed significant covariance with EOAD at chr11:59620206-61870732.. 26](#_Toc185500156)

Note: All base-pair positions in the Figs are reported using genome build GRCh37.

# Fig A. Locus zoom plots showing a 1MB region surrounding the EOAD top SNP chr19:45396665 and any overlapping genome-wide significant loci from the lipids GWAS.

1. EOAD


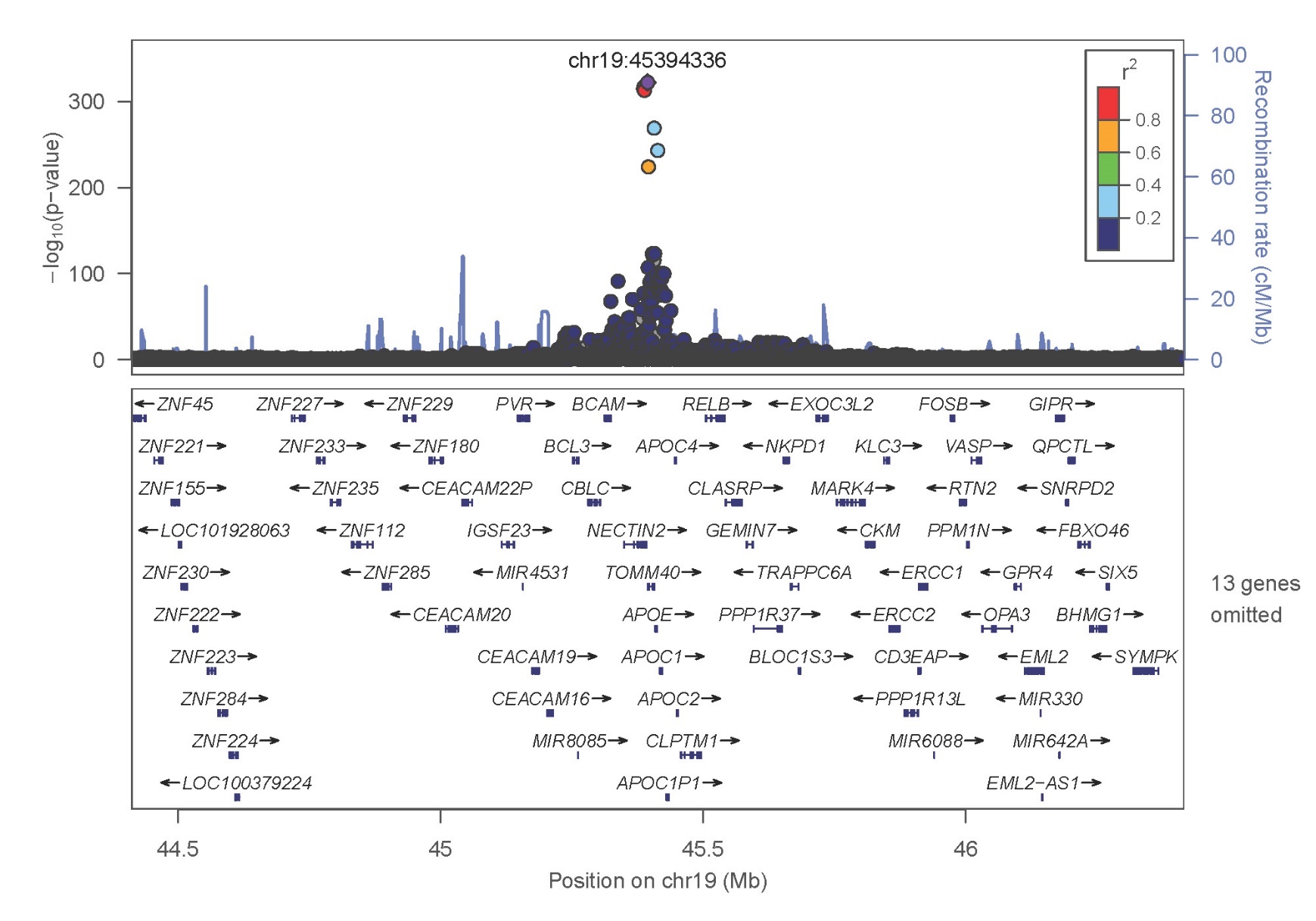


1. HDL


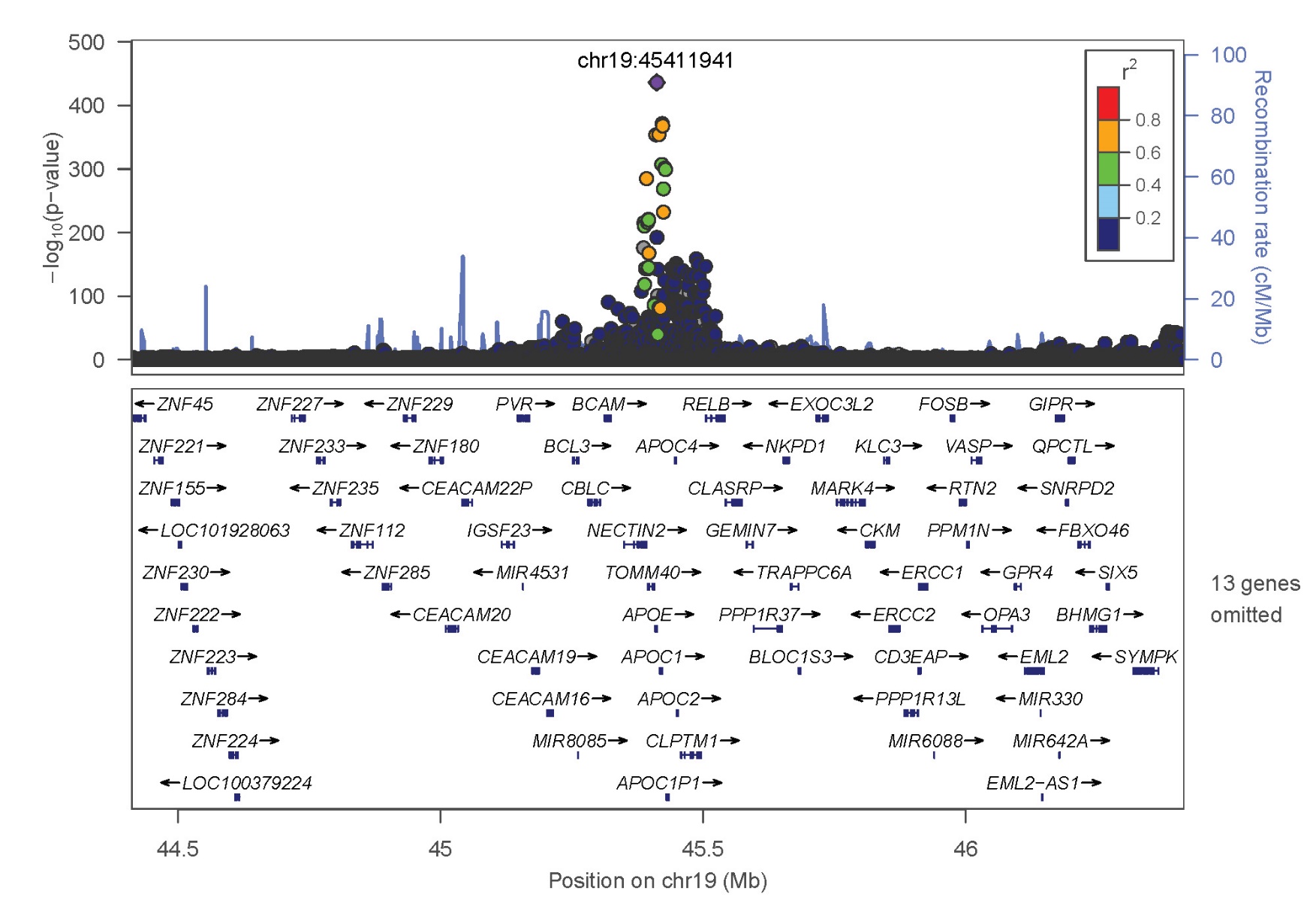


1. LDL


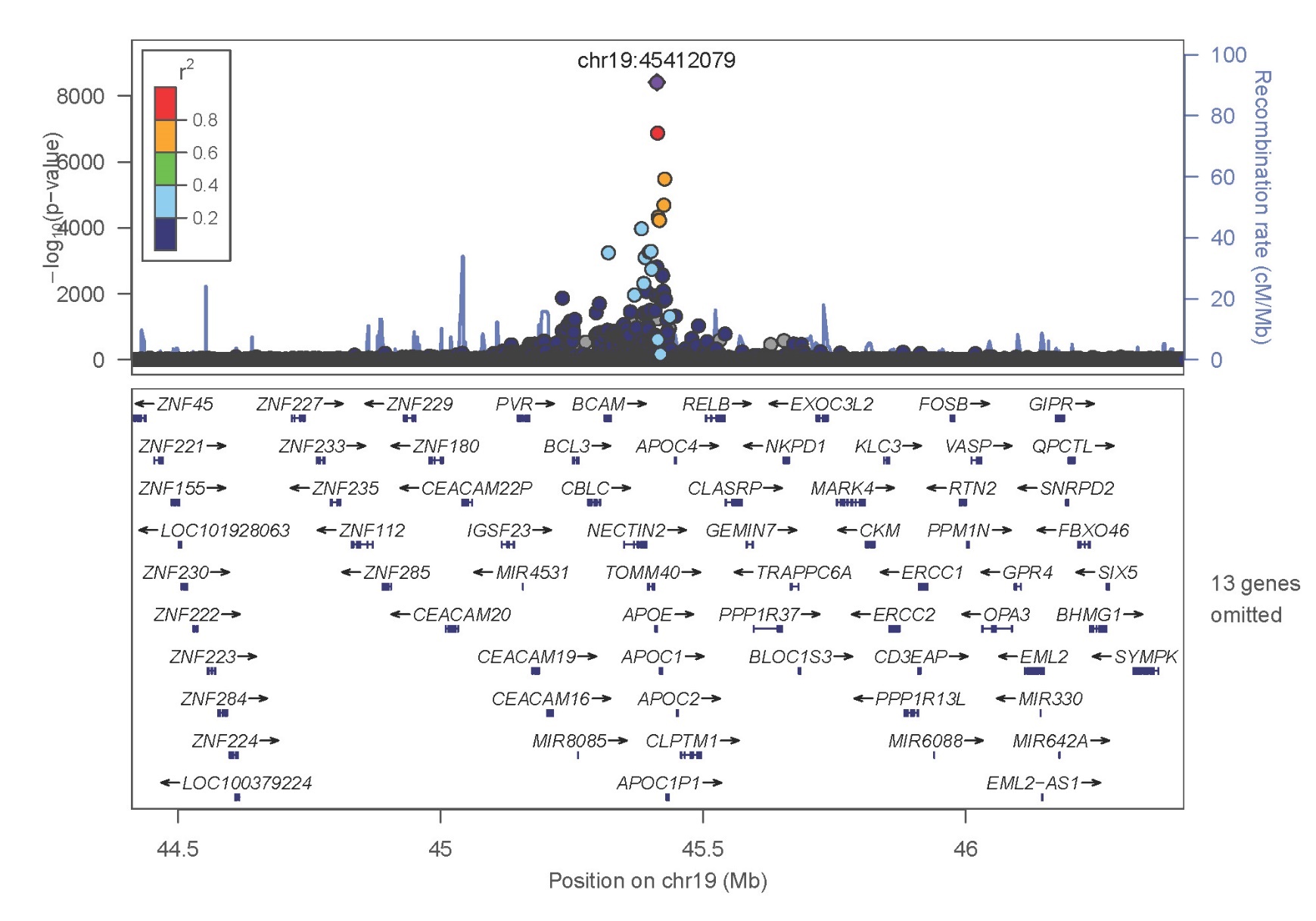


1. nonHDL


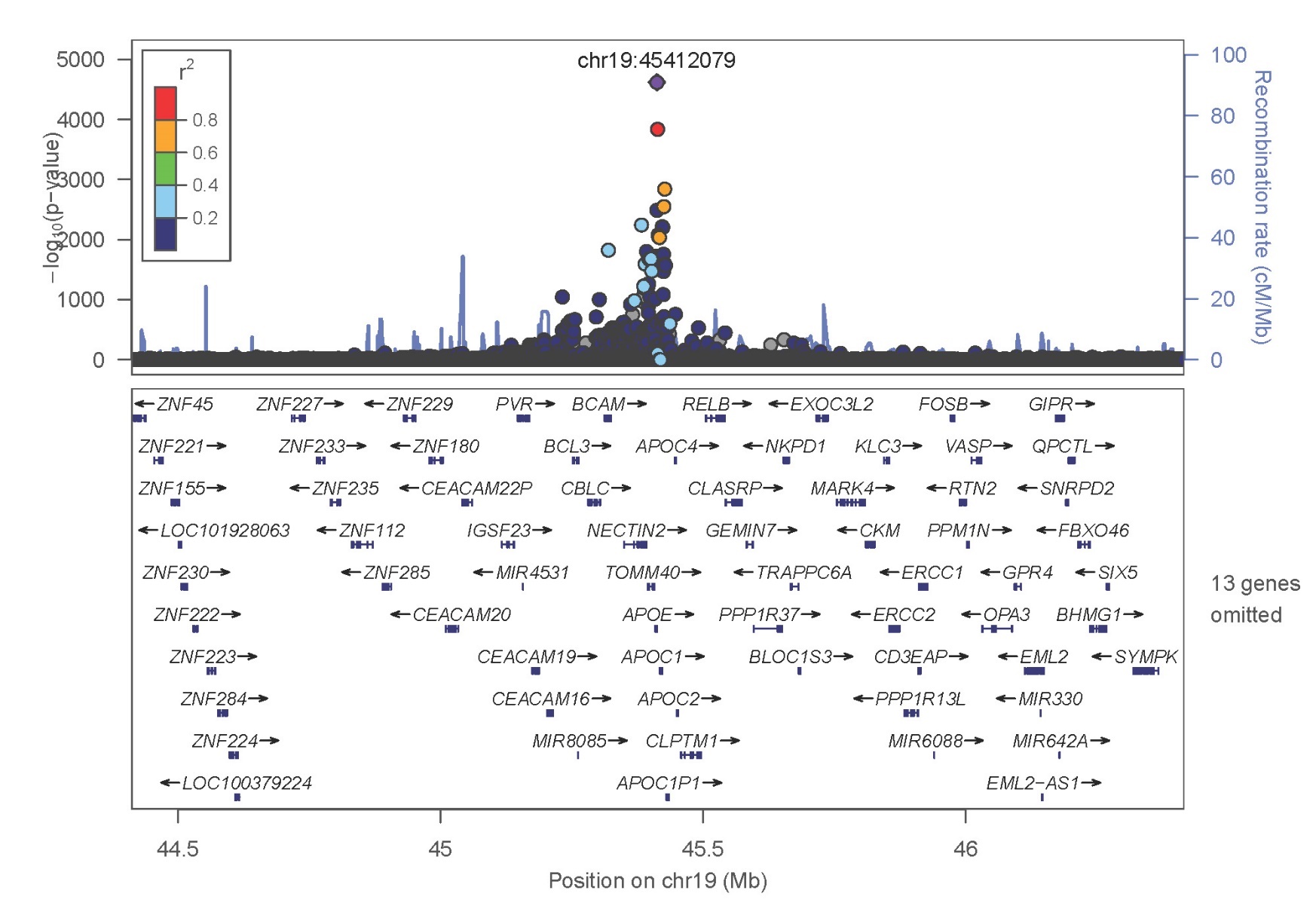


1. TC


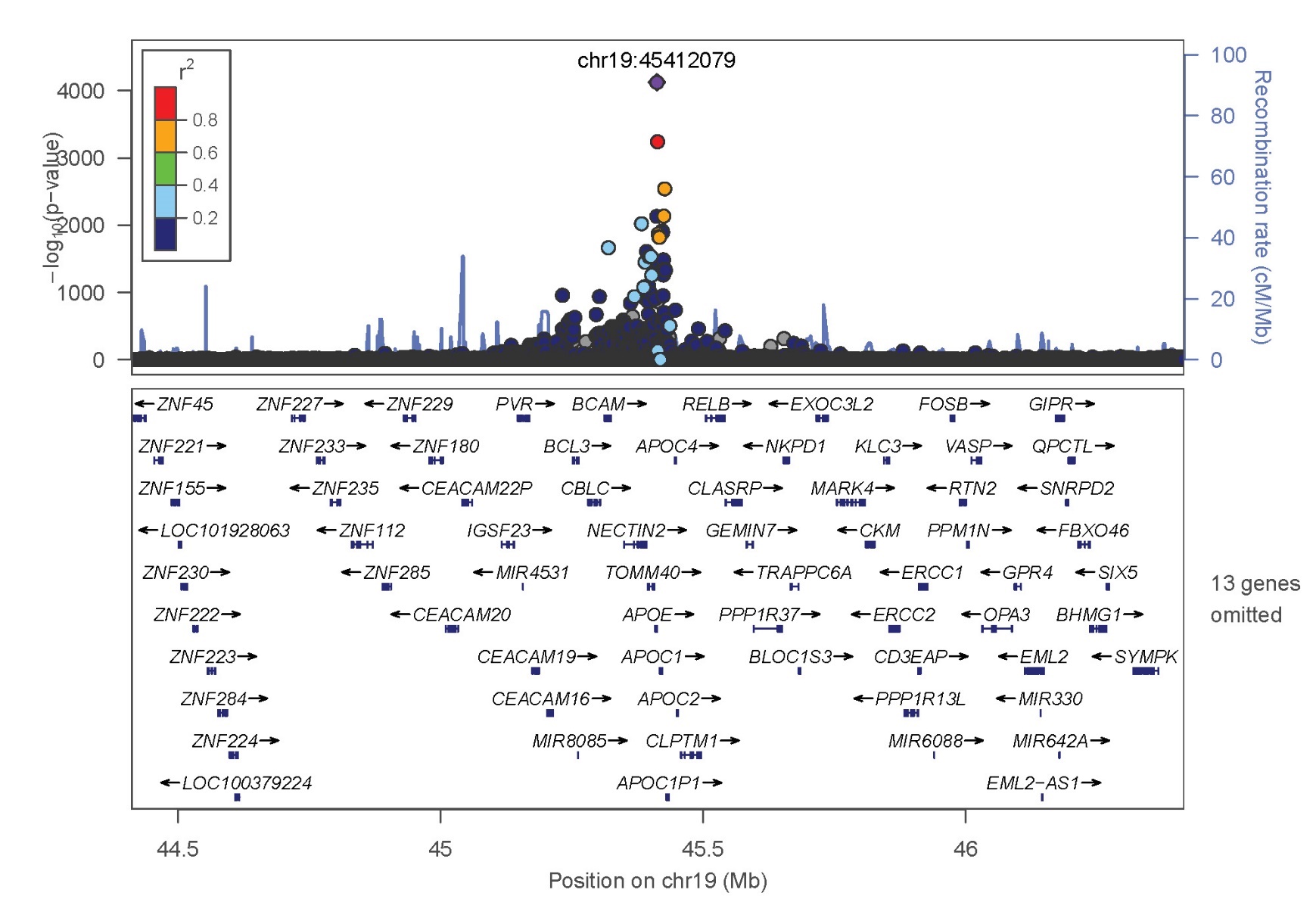


1. TG


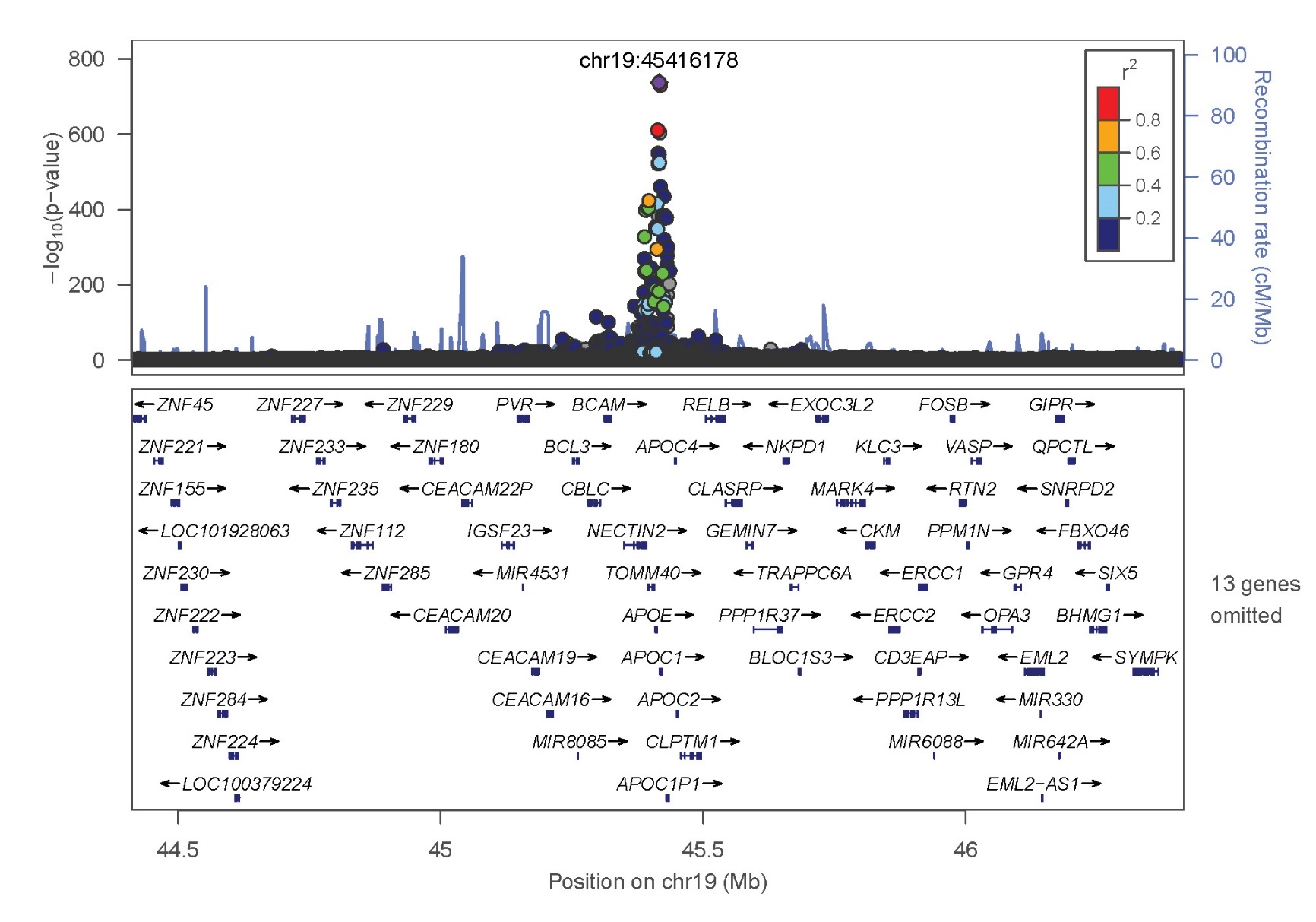


# Fig B. Locus zoom plots showing a 1MB region surrounding the EOAD top SNP chr6:41129252 and any overlapping genome-wide significant loci from the lipids GWAS.

1. EOAD


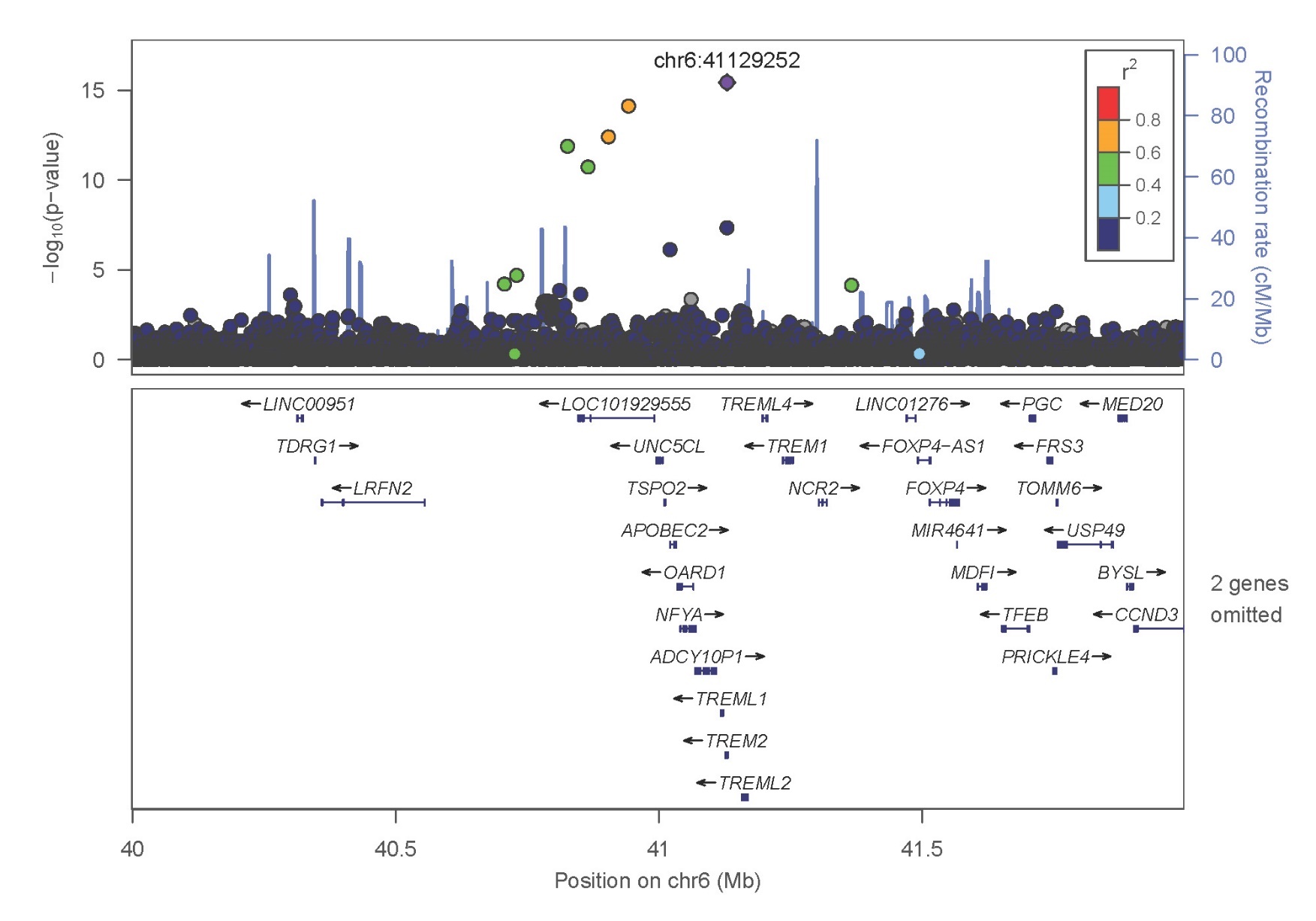


1. TG


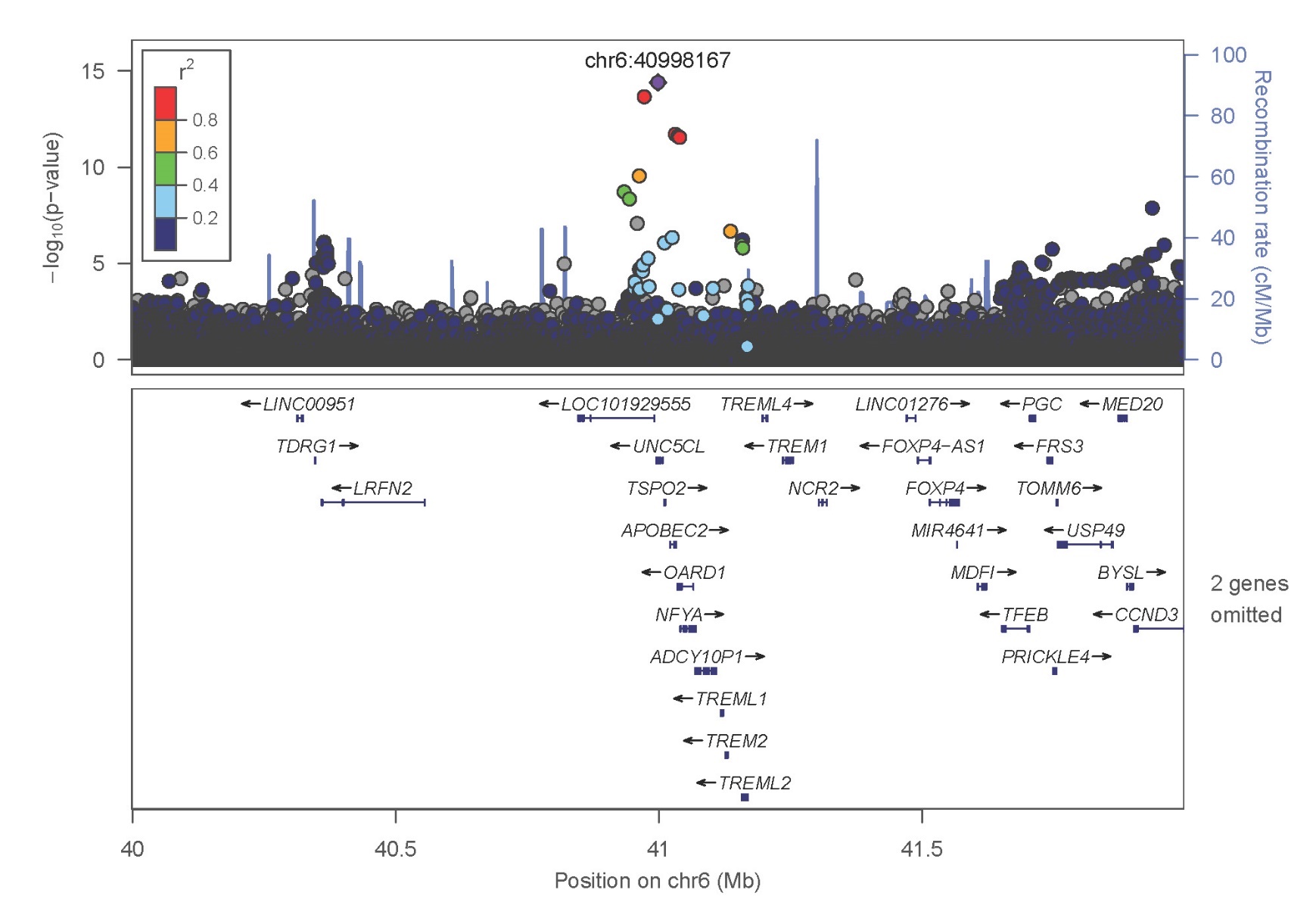


# Fig C. Locus zoom plots showing a 1MB region surrounding the EOAD top SNP chr11:60076693 and any overlapping genome-wide significant loci from the lipids GWAS.

1. EOAD


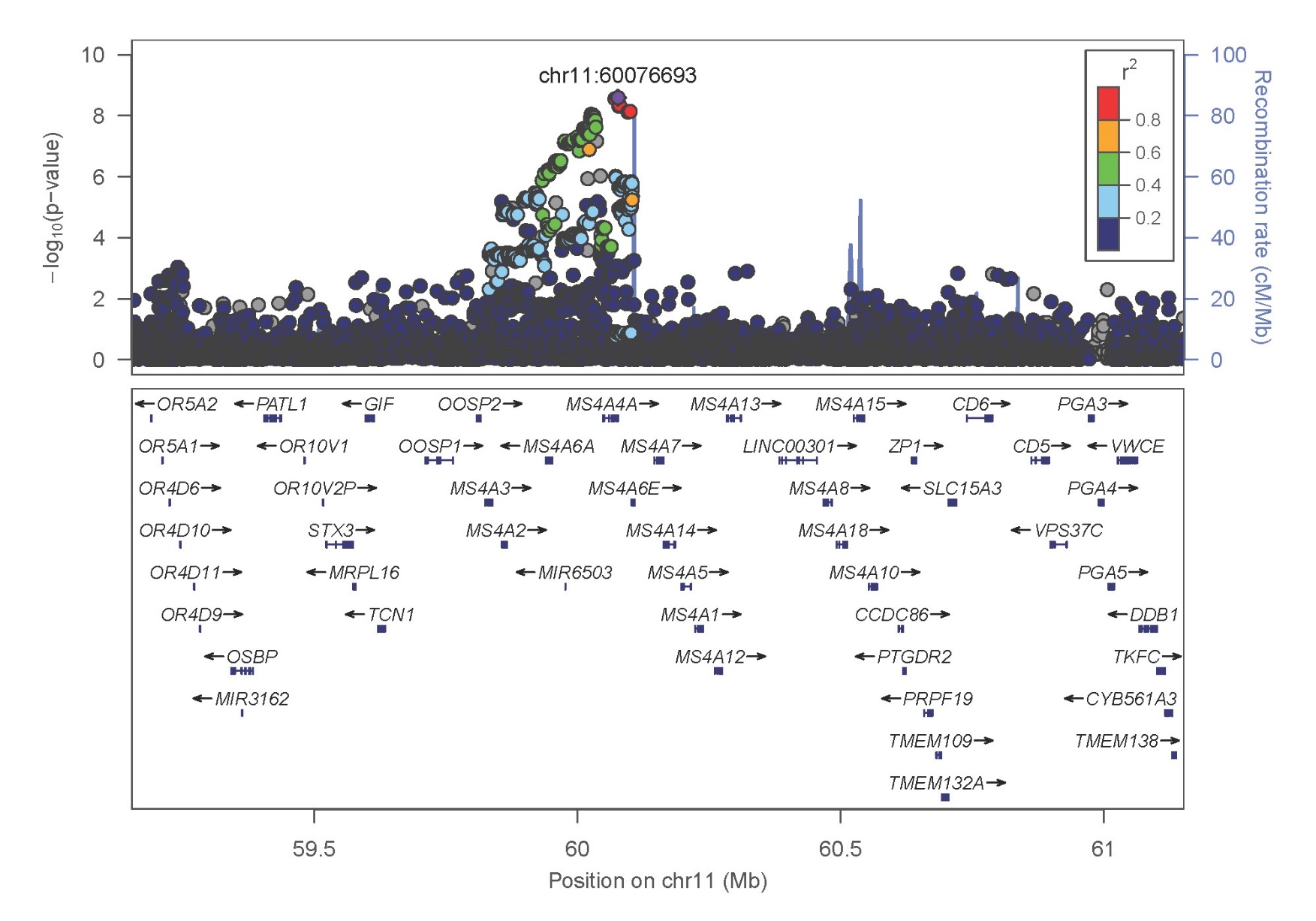


1. TC


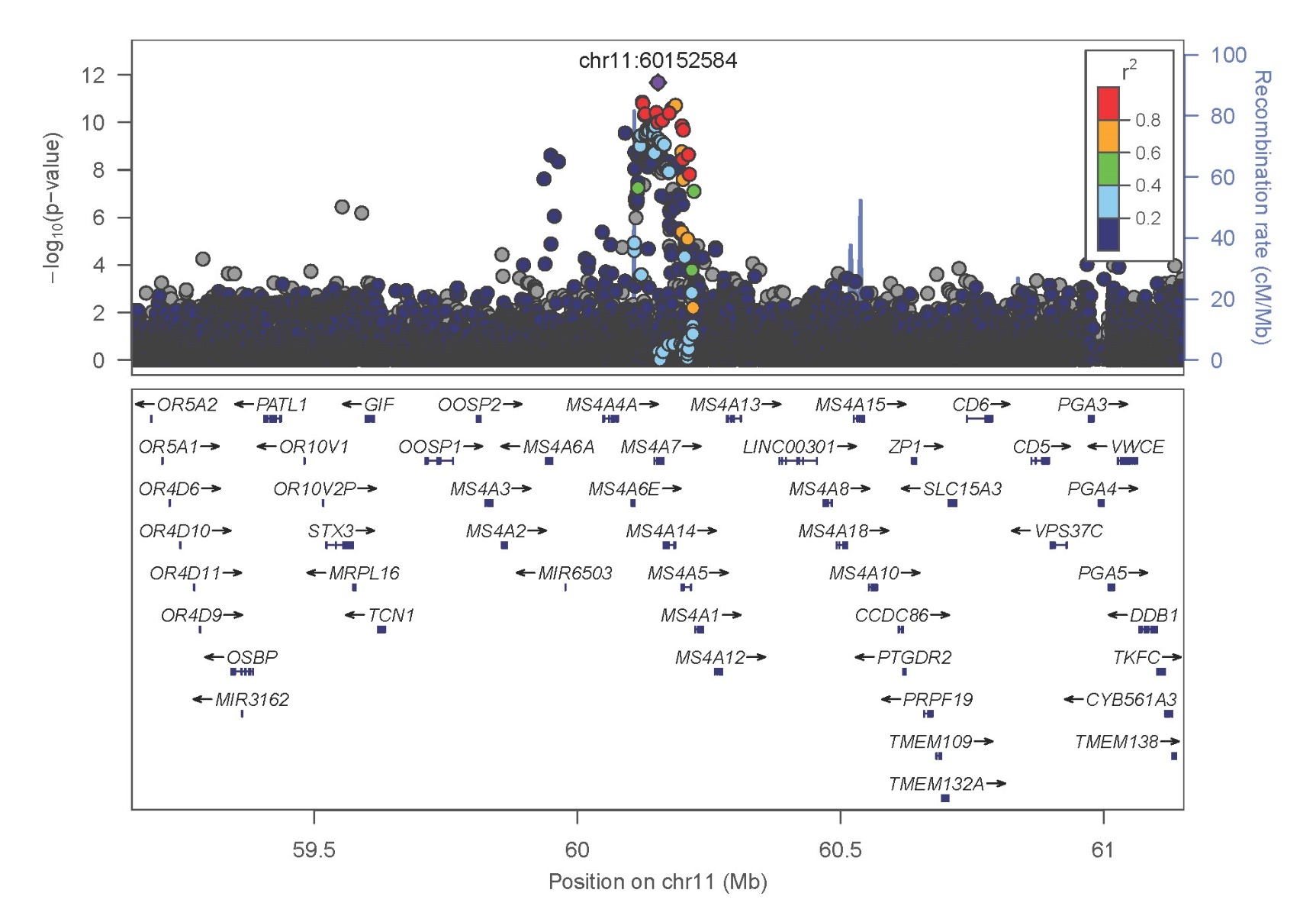


# Fig D. Locus zoom plots showing a 1MB region surrounding the EOAD top SNP chr19:54814234 and any overlapping genome-wide significant loci from the lipids GWAS.

1. EOAD


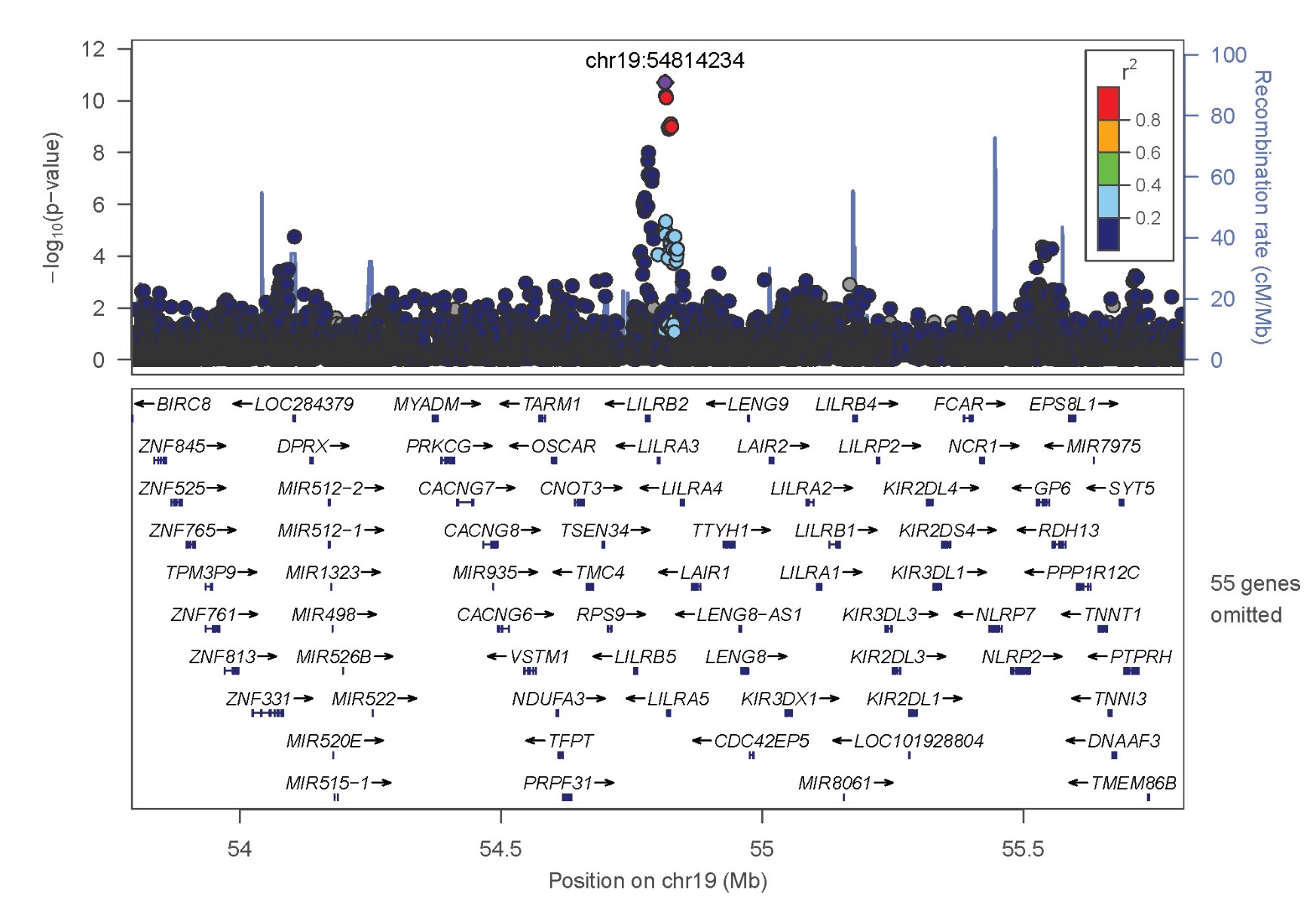


1. HDL


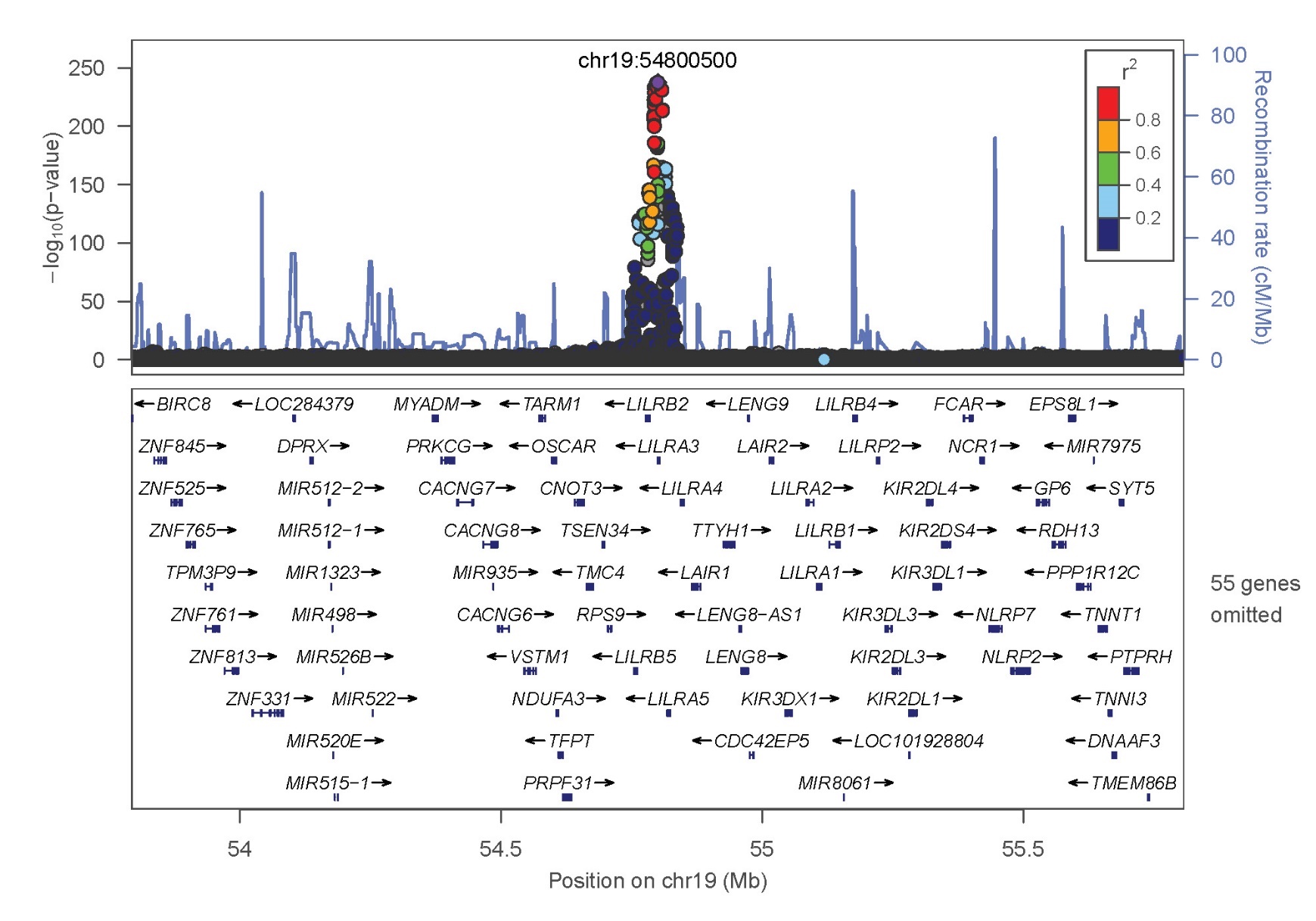


1. TC


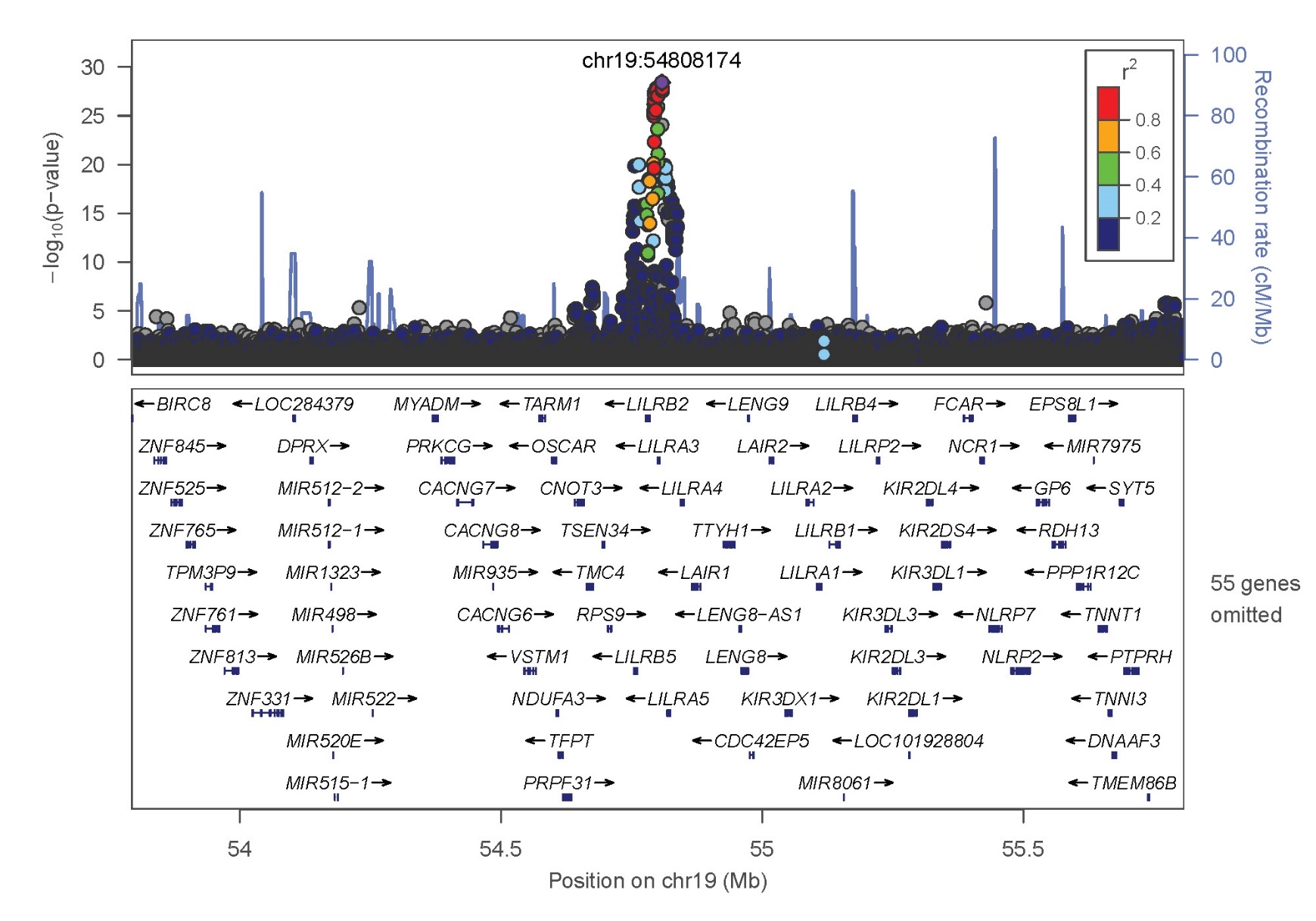


1. TG


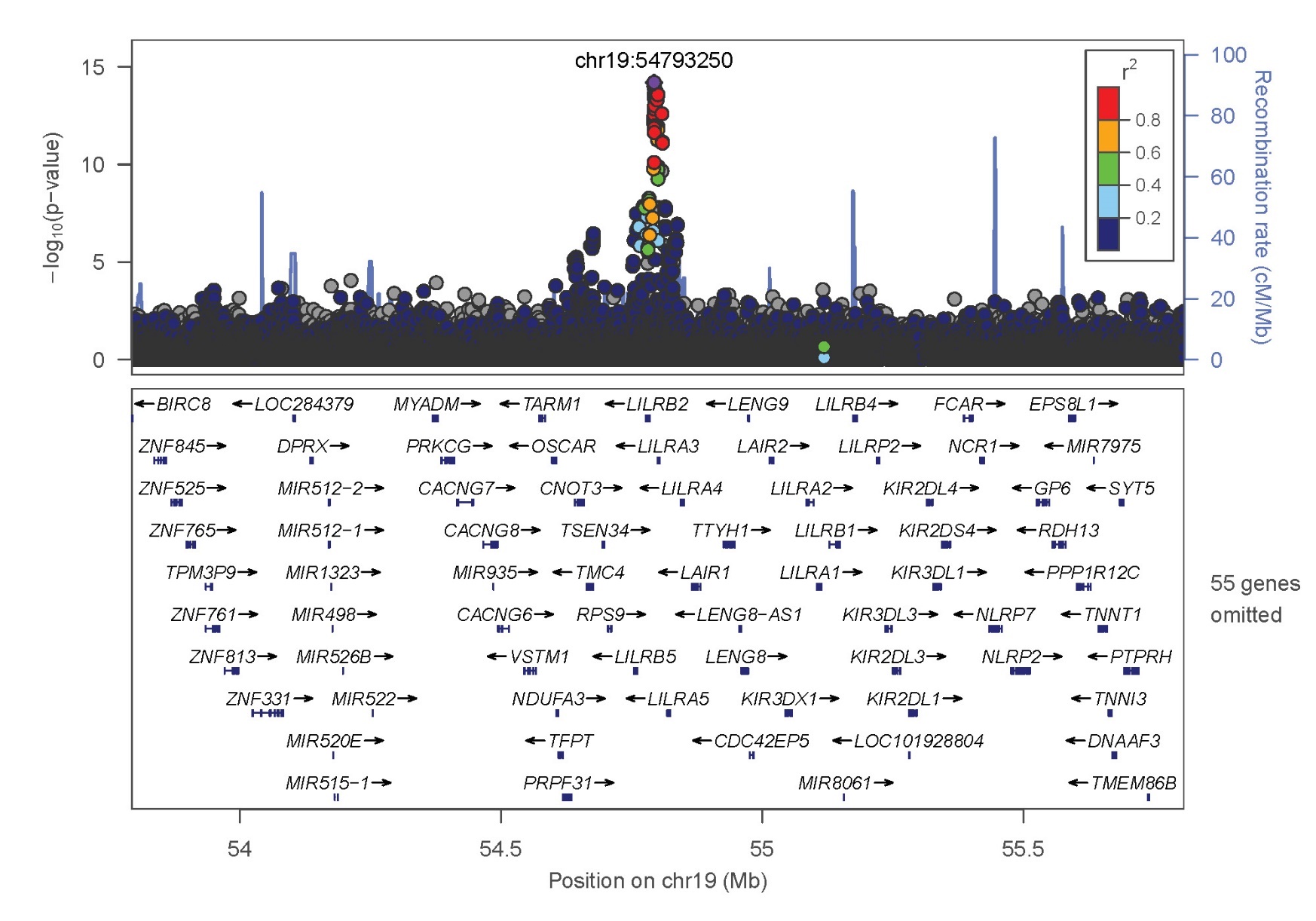


# Fig E. Locus zoom plots showing a 1MB region surrounding the EOAD top SNP chr19:18533642 and any overlapping genome-wide significant loci from the lipids GWAS.

1. EOAD


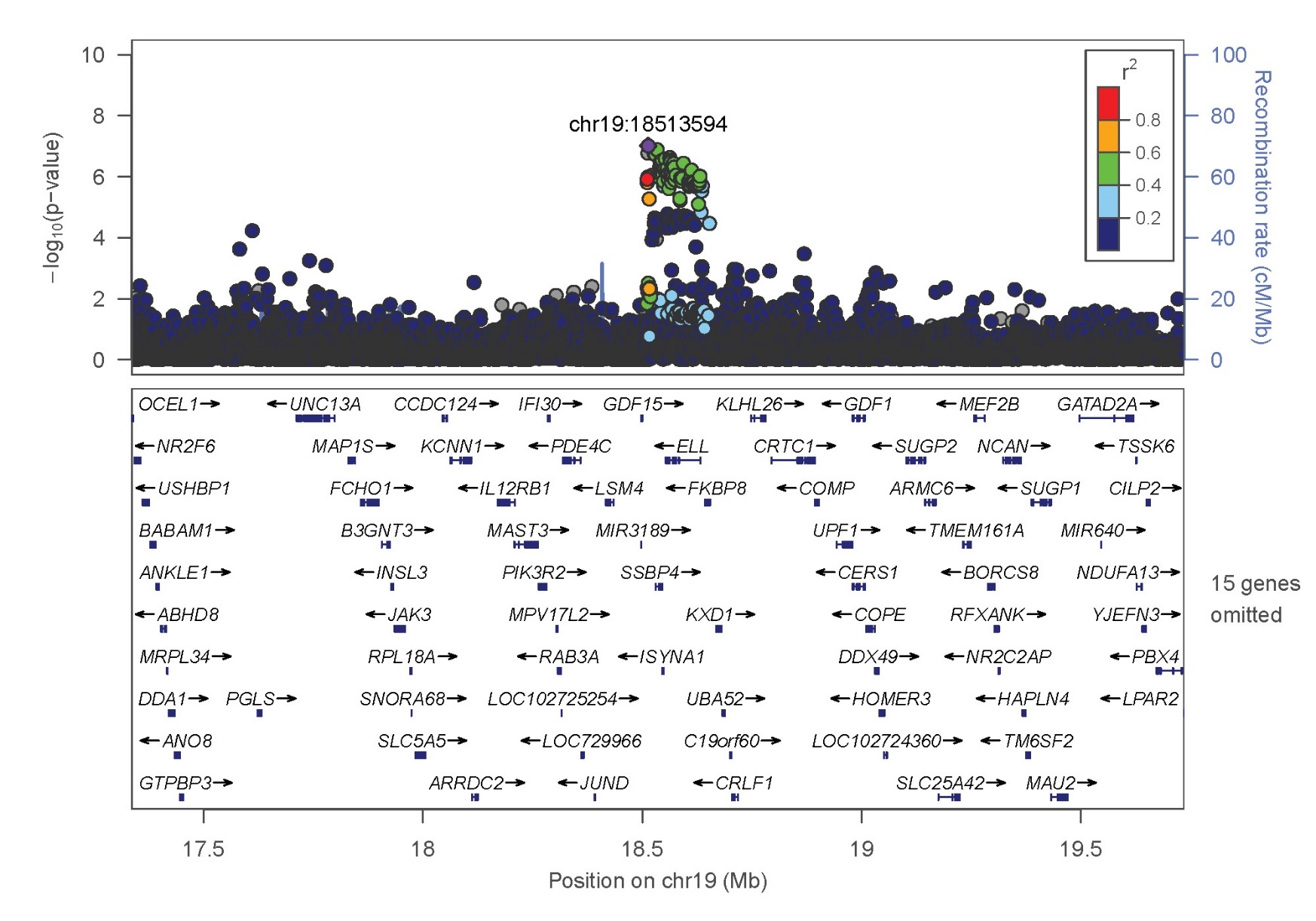


1. HDL


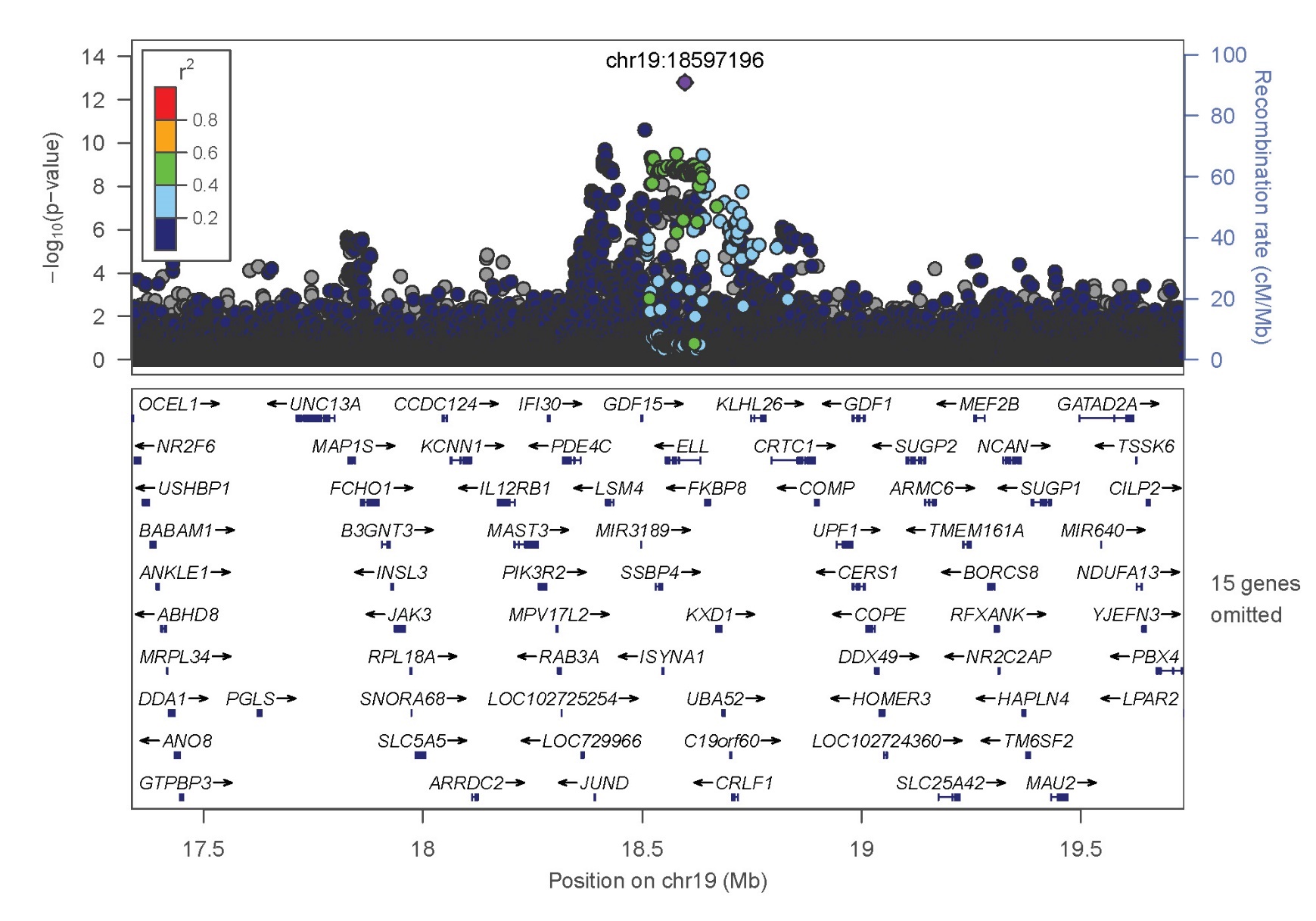


# Fig F. Locus zoom plots for EOAD and each lipid trait that showed significant covariance with EOAD at chr5:73508509-75240469.

1. EOAD


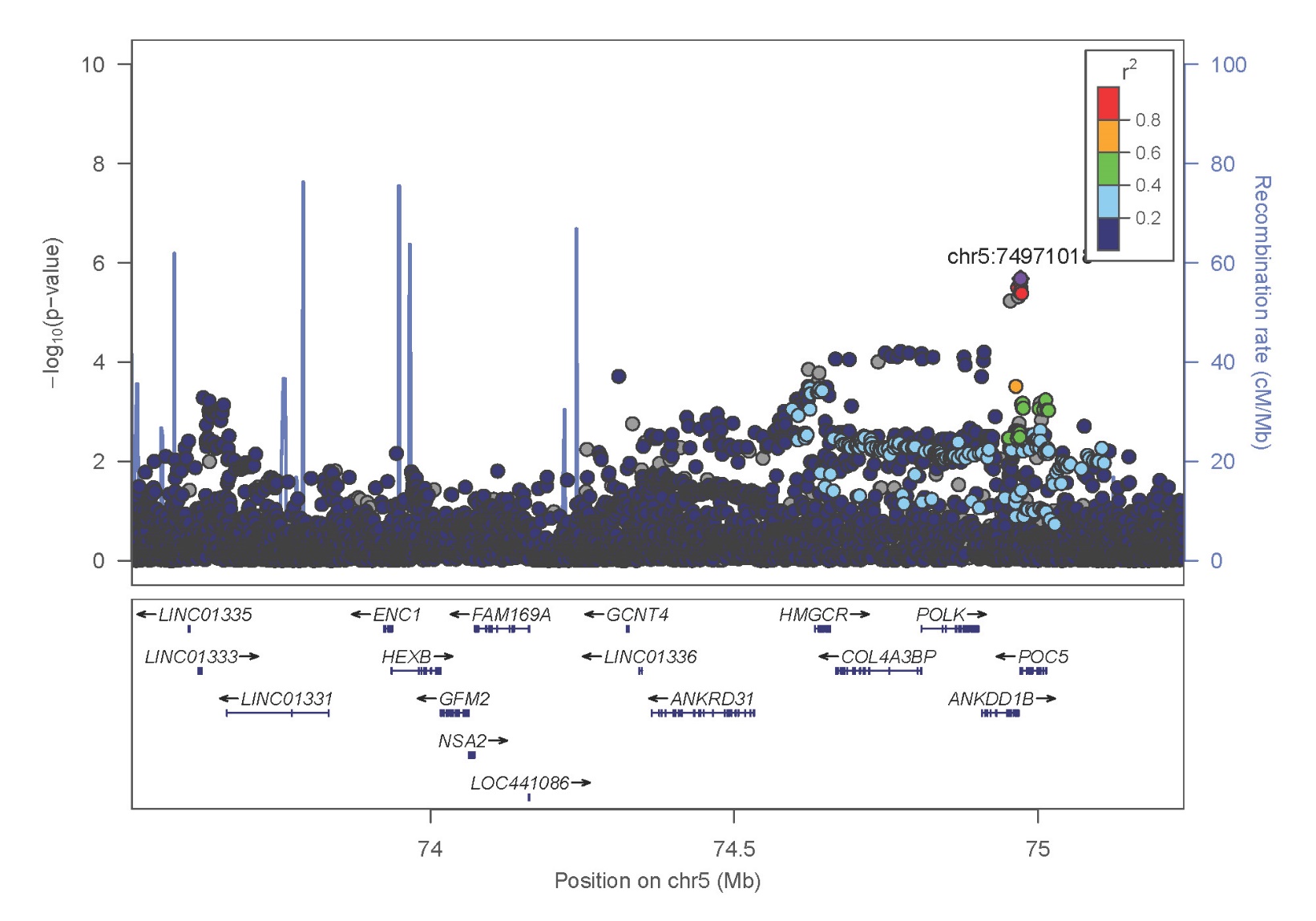


1. TC


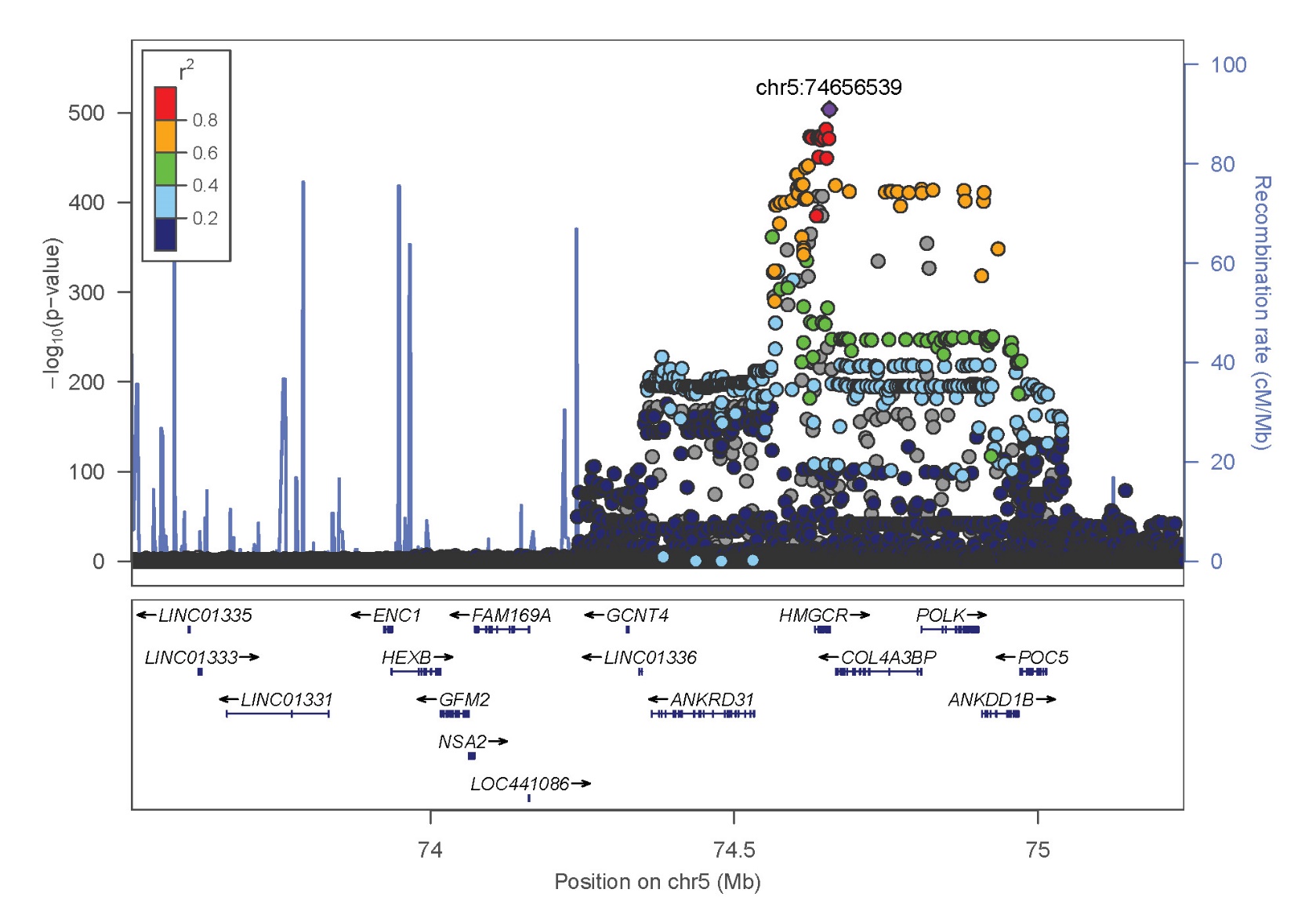


1. LDL-C


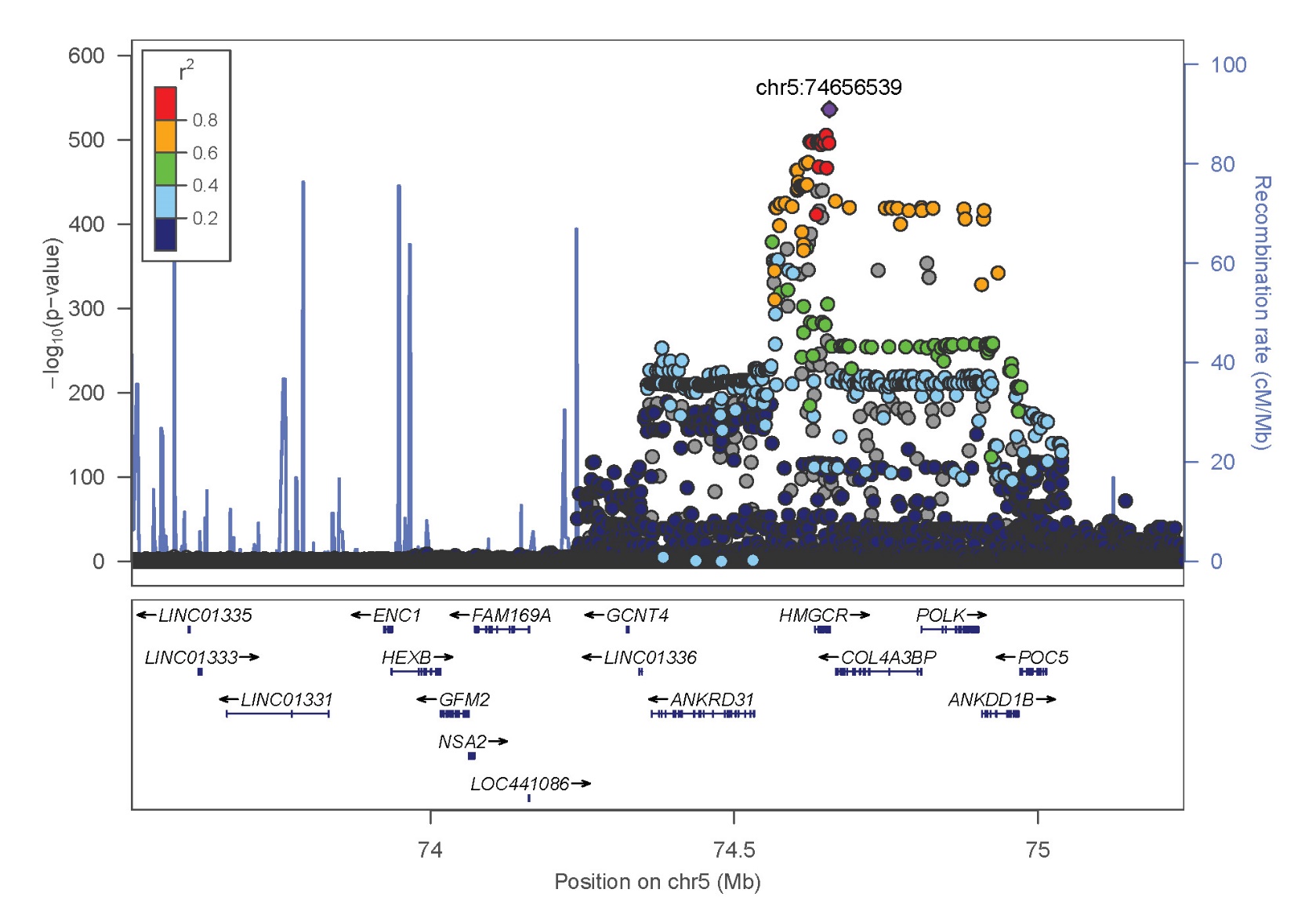


1. nonHDL-C


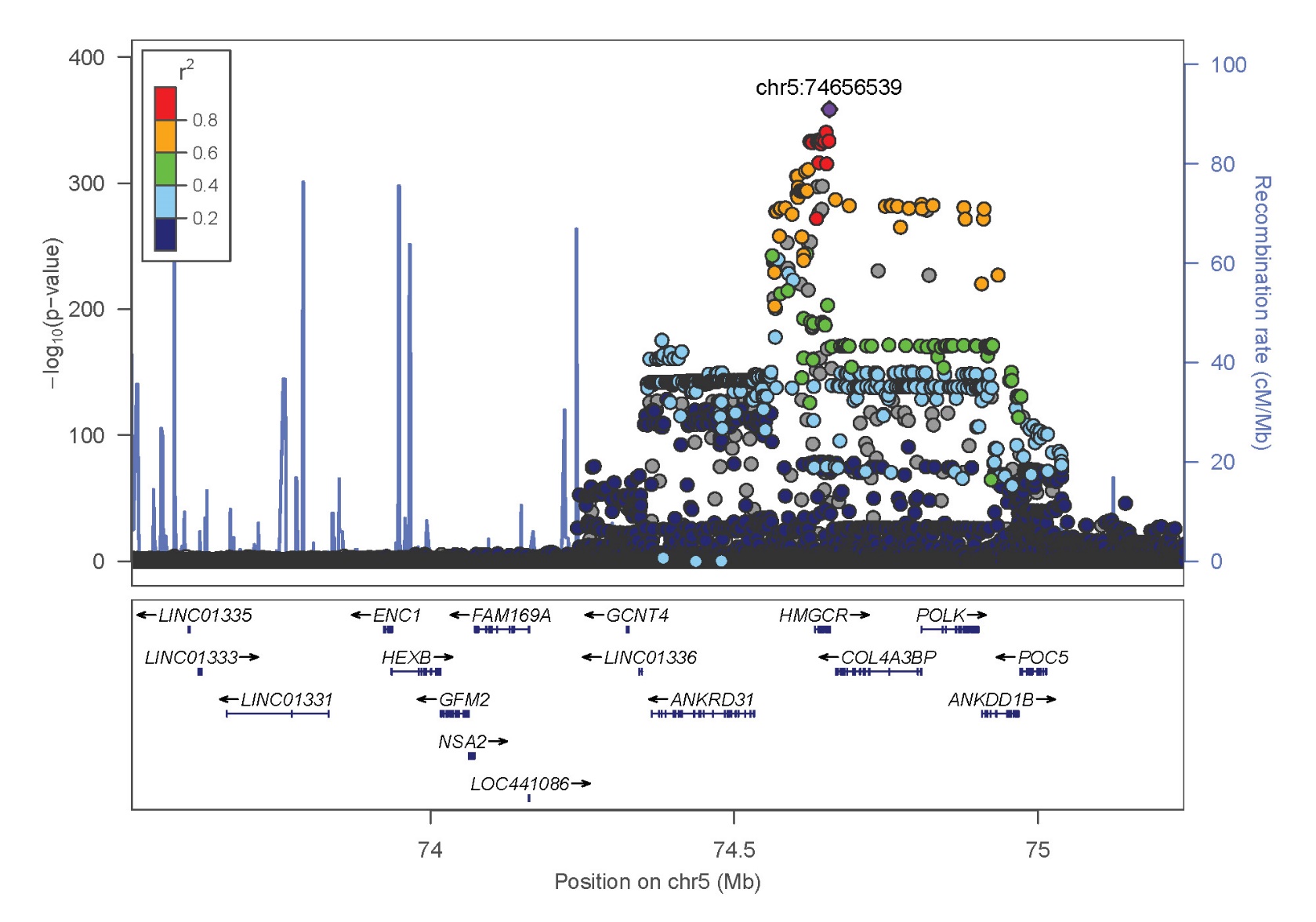


# Fig G. Locus zoom plots for EOAD and each lipid trait that showed significant covariance with EOAD at chr10:123855124-124894743.

1. EOAD


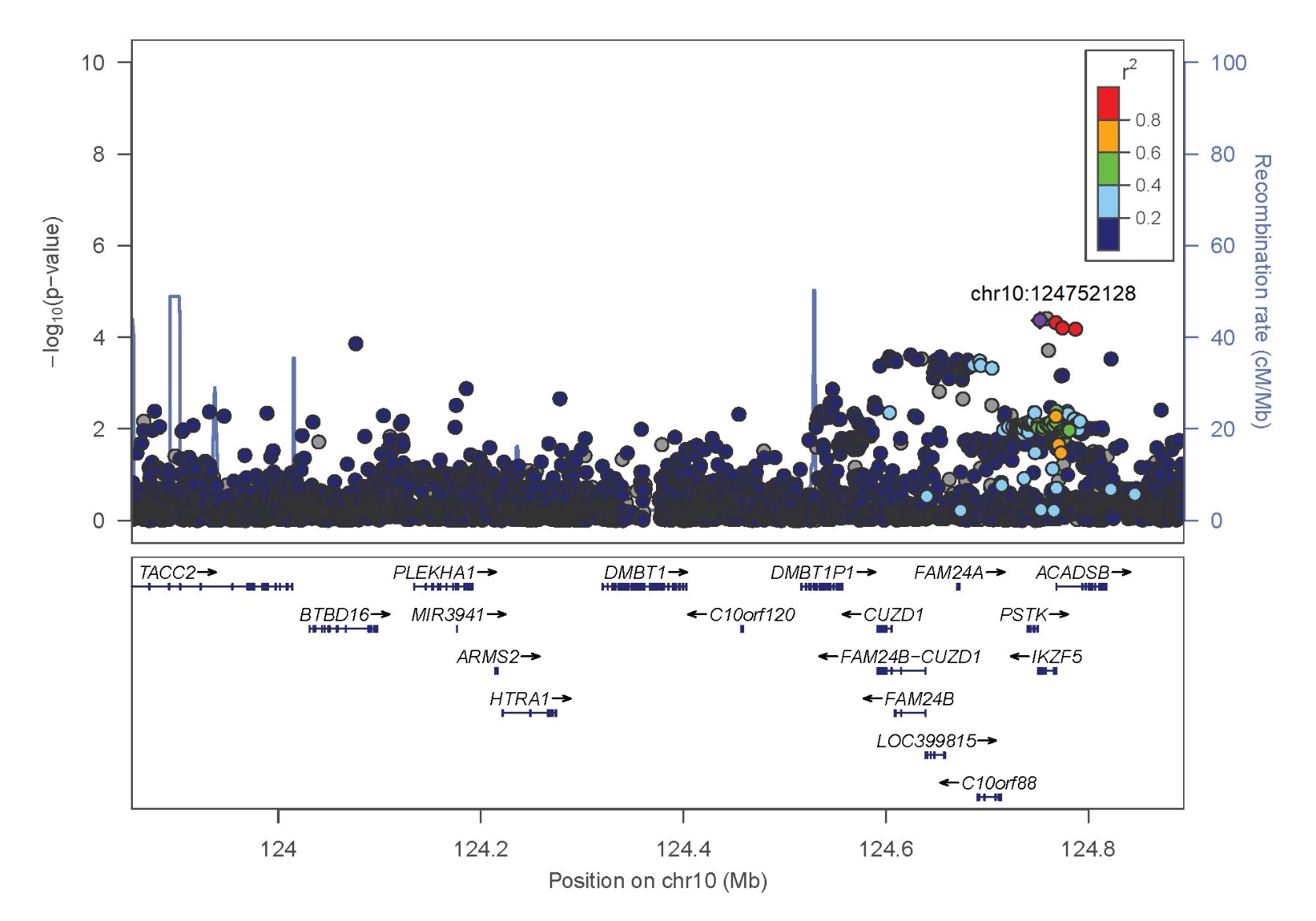


Note: Reference SNP was changed to second lowest P-value because the SNP with the lowest P-value did not appear in the 1000 Genomes reference panel and therefore did not show LD information.

1. TC


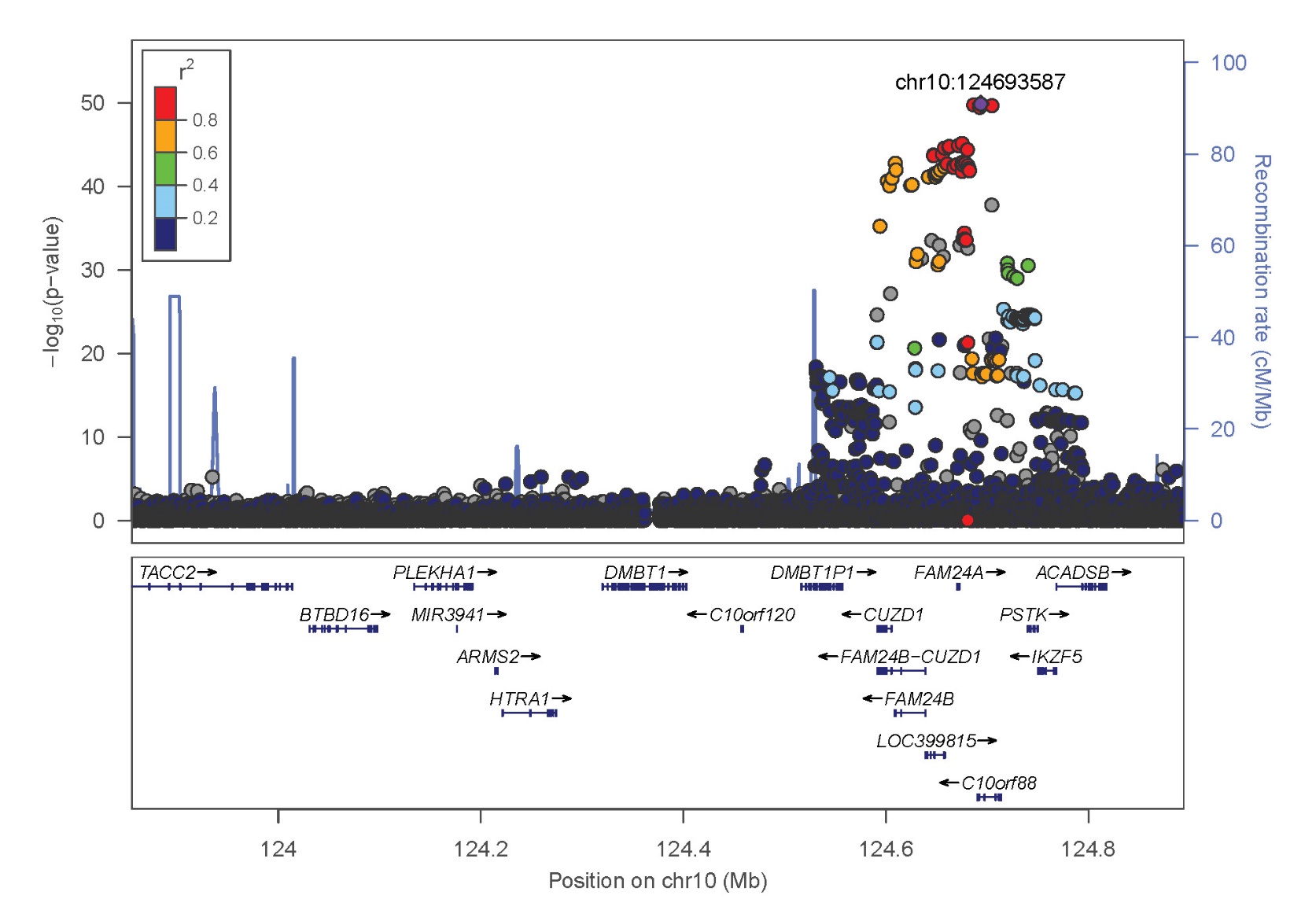


1. LDL-C


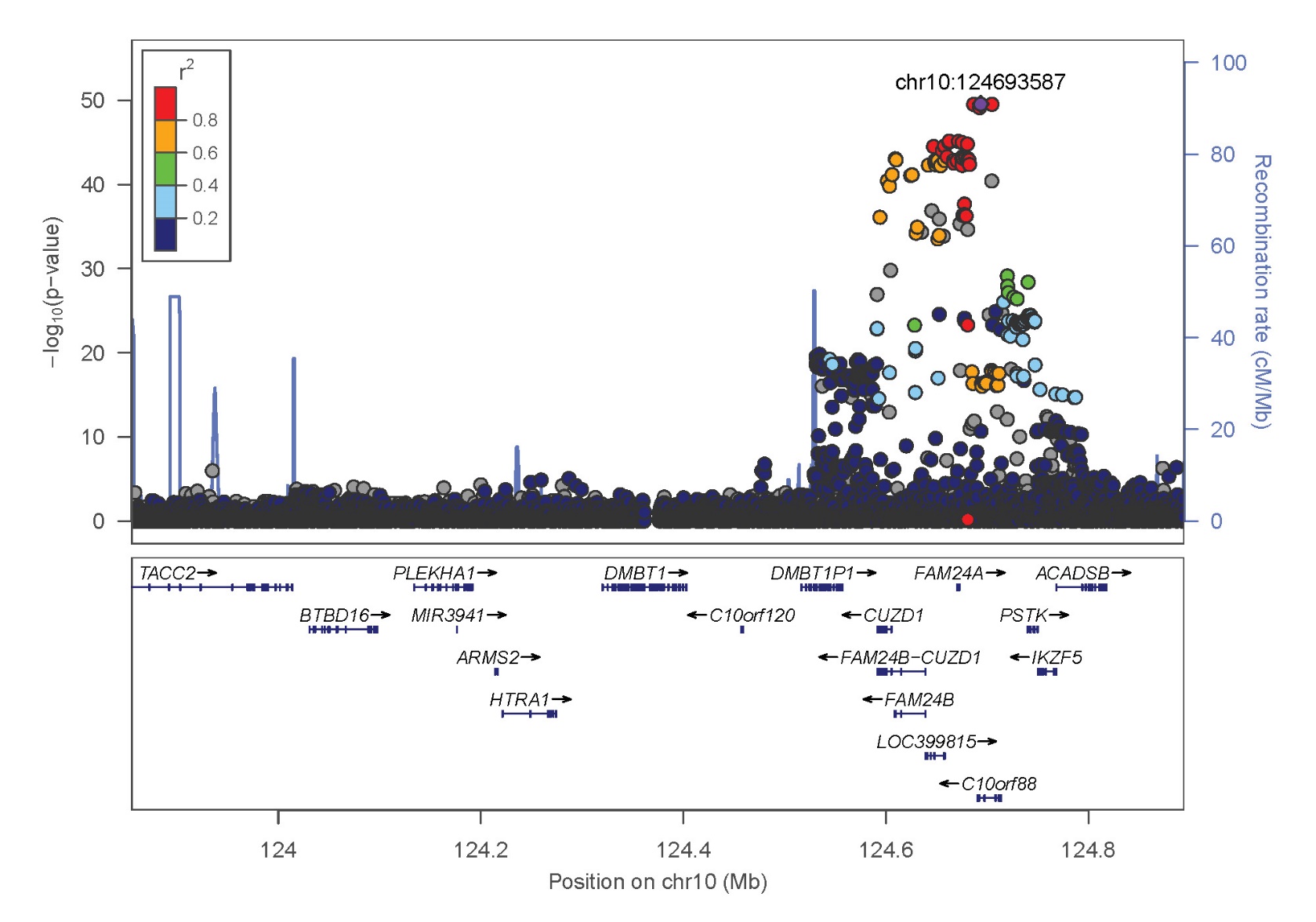


1. nonHDL-C


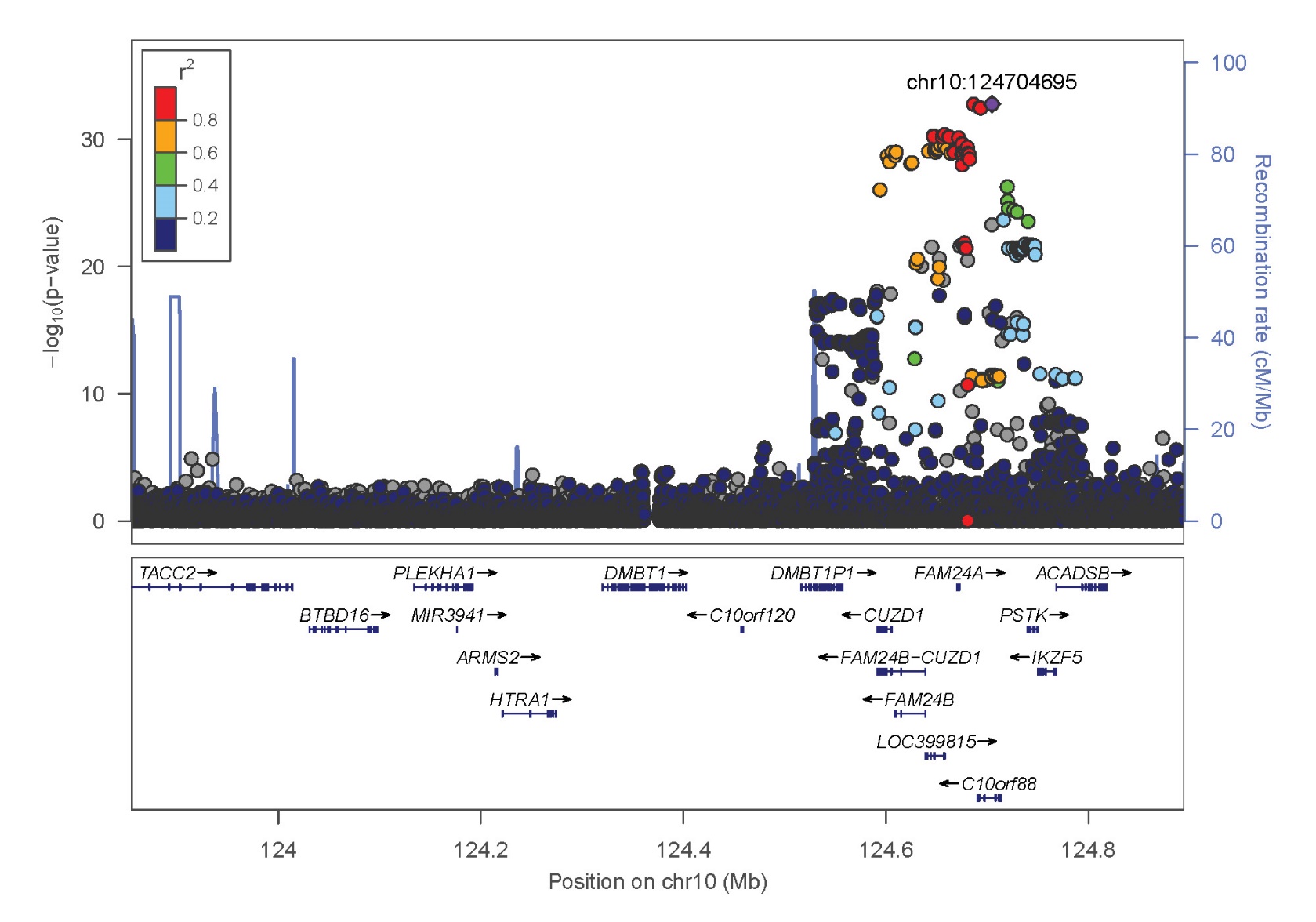


# Fig H. Locus zoom plots for EOAD and each lipid trait that showed significant covariance with EOAD at chr11:59620206-61870732. Because of the extremely large effect shown to the right side of the nonHDL-C plot increasing the scale of the y-axis, we also include a plot for that trait zoomed into the region under the peak of the EOAD top hit.

1. EOAD


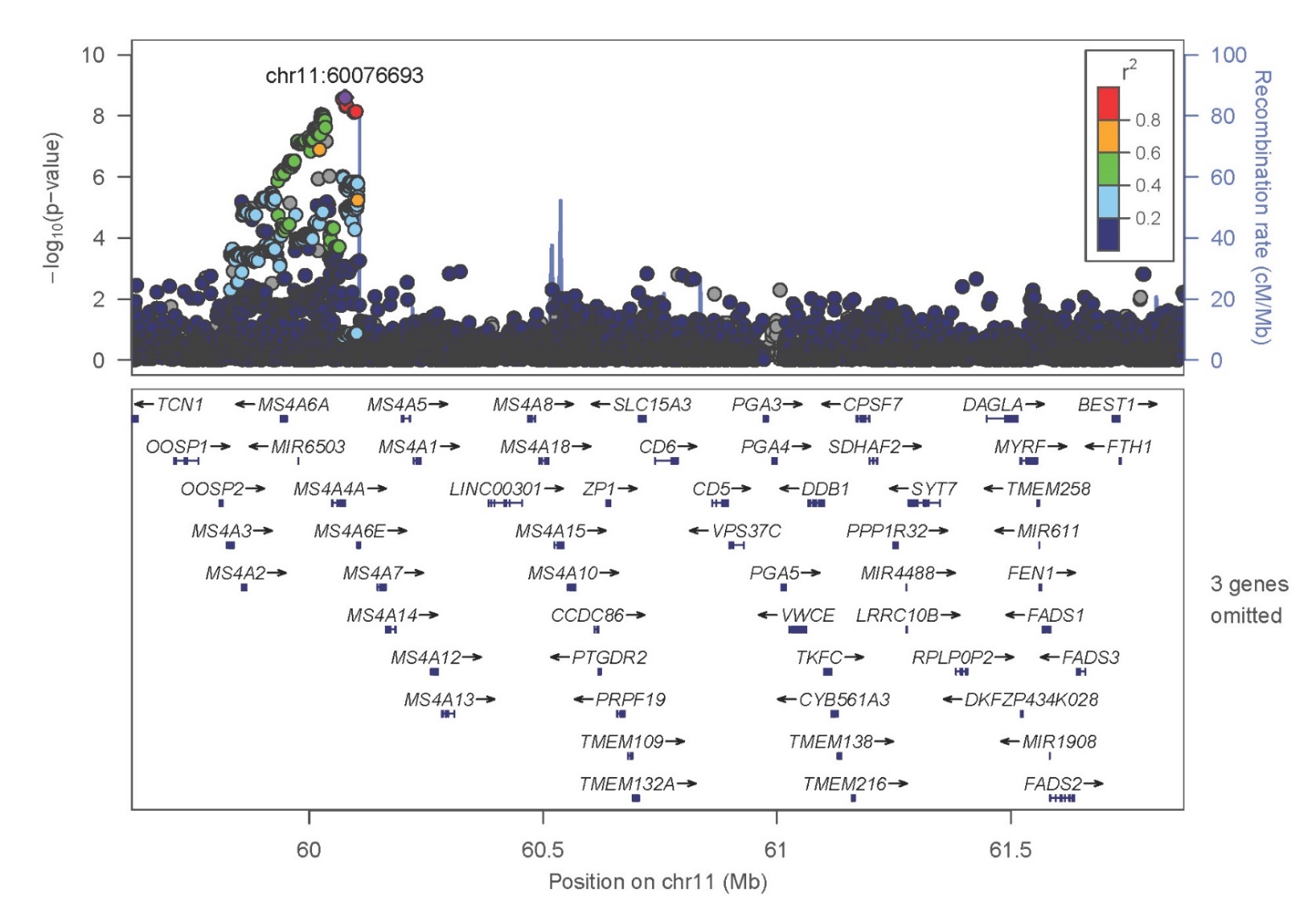


1. nonHDL-C


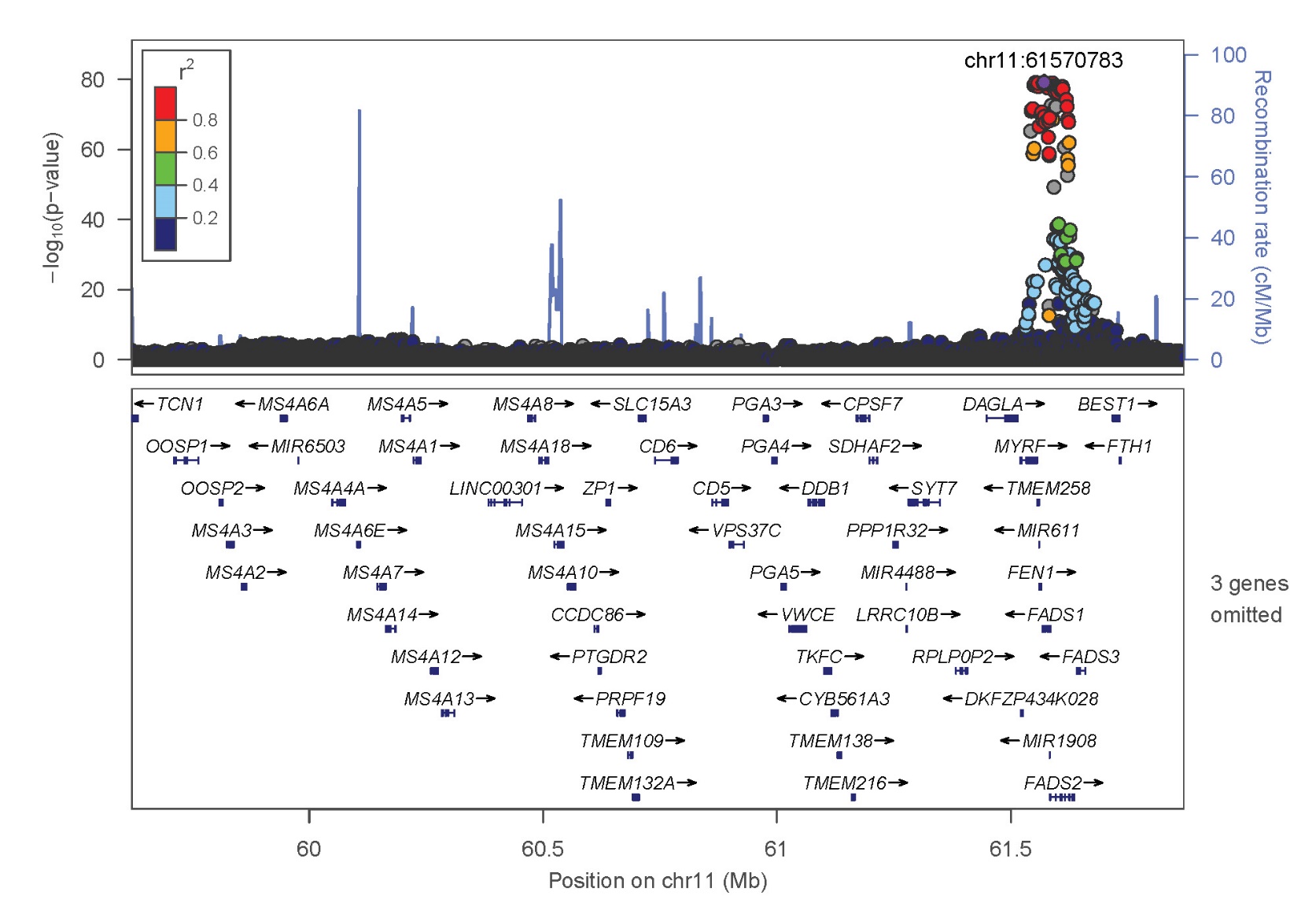


1. nonHDL-C zoomed into the region under the peak of the EOAD top hit


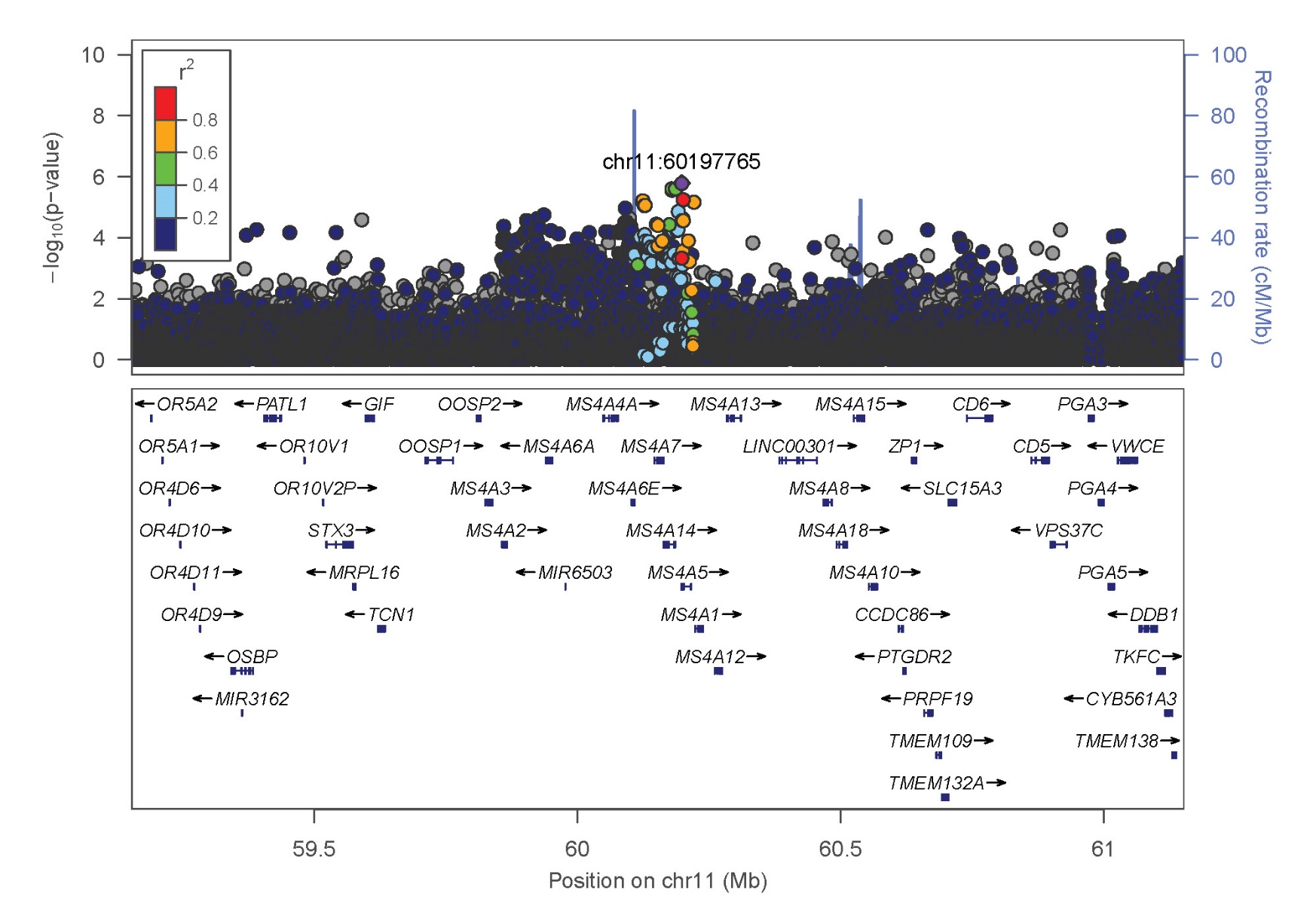

Supplement: S1 Text — Fig A. Locus zoom plots showing a 1MB region surrounding the EOAD top SNP chr19:45396665 and any overlapping genome-wide significant loci from the lipids GWAS. Fig B. Locus zoom plots showing a 1MB region surrounding the EOAD top SNP chr6:41129252 and any overlapping genome-wide significant loci from the lipids GWAS. Fig C. Locus zoom plots showing a 1MB region surrounding the EOAD top SNP chr11:60076693 and any overlapping genome-wide significant loci from the lipids GWAS. Fig D. Locus zoom plots showing a 1MB region surrounding the EOAD top SNP chr19:54814234 and any overlapping genome-wide significant loci from the lipids GWAS. Fig E. Locus zoom plots showing a 1MB region surrounding the EOAD top SNP chr19:18533642 and any overlapping genome-wide significant loci from the lipids GWAS. Fig F. Locus zoom plots for EOAD and each lipid trait that showed significant covariance with EOAD at chr5:73508509-75240469. Fig G. Locus zoom plots for EOAD and each lipid trait that showed significant covariance with EOAD at chr10:123855124-124894743. In Fig G-i the reference SNP was changed to second lowest P-value because the SNP with the lowest P-value did not appear in the 1000 Genomes reference panel and therefore did not show LD information. Fig H. Locus zoom plots for EOAD and each lipid trait that showed significant covariance with EOAD at chr11:59620206-61870732. Because of the extremely large effect shown to the right side of the nonHDL-C plot increasing the scale of the y-axis, we also include a plot for that trait zoomed into the region under the peak of the EOAD top hit. (DOCX) [file pgen.1011631.s001.docx]
